# Early-life nutritional supplementation protects against home environmental risks in Ghanaian children's social-emotional development

**DOI:** 10.3389/fnut.2025.1571677

**Published:** 2025-08-20

**Authors:** Lindsey C. Partington, Haiying Yuan, Maku E. Demuyakor, Seth Adu-Afarwuah, Amanda E. Guyer, Kathryn G. Dewey, Sika M. Kumordzie, Brietta M. Oaks, Charles D. Arnold, Elizabeth L. Prado, Paul D. Hastings

**Affiliations:** ^1^Institute for Global Nutrition, University of California, Davis, Davis, CA, United States; ^2^Center for Mind & Brain, University of California, Davis, Davis, CA, United States; ^3^Texas Health and Human Services Commission, Austin, TX, United States; ^4^McKing Consulting Corporation, Atlanta, GA, United States; ^5^Department of Nutrition and Food Science, University of Ghana, Accra, Ghana; ^6^Department of Human Ecology, University of California, Davis, Davis, CA, United States; ^7^Department of Nutrition, University of Rhode Island, Kingston, RI, United States; ^8^Department of Psychology, University of California, Davis, Davis, CA, United States

**Keywords:** nutritional supplement, social-emotional development, home environment, low-and middle-income countries, integrated intervention, small-quantity lipid-based nutrient supplement

## Abstract

**Introduction:**

This study examines how an early-life small-quantity lipid-based nutrient supplement (LNS) interacts with the home environment to influence Ghanaian children's social-emotional functioning at preschool age.

**Method:**

In a randomized controlled trial, 1,320 Ghanaian women received either daily LNS, multiple micronutrients (MMN) or iron and folic acid (IFA) during pregnancy through 6 months postpartum. Infants in the LNS group received the daily supplement from 6 to 18 months. The IFA and MMN groups were combined to be the control group. At 4–6 years (*n* = 966), we assessed children's home environments (caregiver responsivity, negative behavior acceptance, physical environment, learning materials, academic stimulation) and their social-emotional strengths and difficulties.

**Results:**

Both LNS and higher quality home environments—particularly caregiver responsivity and negative behavior acceptance—predicted children having fewer social-emotional difficulties. LNS supplementation moderated relations between the home environment and children's hyperactivity/inattention and total difficulties. Living in a safer physical environment was related to fewer hyperactivity/inattention problems and total difficulties only for children who received LNS. Less academic stimulation was related to more hyperactivity/inattention only for children in the control group.

**Conclusion:**

Early-life LNS supplementation and supportive home environments reduce social-emotional difficulties in Ghanaian children. Nutritional interventions are most effective in the context of safe, stimulating households, demonstrating that integrated interventions support children's wellbeing in low- and middle-income countries.

**Clinical trial registration:**

clinicaltrials.gov; identifier: NCT00970866.

## Introduction

An estimated 250 million children under 5 years of age in low- and middle-income countries (LMICs) are at risk of not fulfilling their developmental potential due to a combination of nutritional, psychosocial, and poverty-related risk factors (1, 2). Approximately 26% of young children in LMICs fail to meet social-emotional milestones by age 4 (3), placing them at heightened risk for poor mental health later in life (4, 5). Children in LMICs are disproportionately exposed to undernutrition (6, 7) and low-quality home environments (e.g., few learning opportunities, physically unsafe structures, lack of responsive caregiving) (1, 8), which together elevate children's social-emotional risk (9). Children with strong social-emotional skills—such as prosocial behavior—can better manage their stress and more effectively engage in learning (10, 11), whereas social-emotional difficulties—such as emotional problems, conduct issues, hyperactivity and peer relationship problems—are associated with poor academic performance and increased mental health risks (12–15). Children's social-emotional difficulties are both prevalent and understudied in LMICs (3). Investigating how nutrition and specific home environment factors jointly influence children's social-emotional development is critical for designing effective interventions in low and middle income settings (5, 16–18).

Animal and human studies demonstrate that both nutritional deficiencies and low-quality, under-stimulating environments disrupt similar neurodevelopmental processes (e.g., reduced dendritic branching, altered synaptic density, and impaired myelination), leading to negative impacts on physical, motor, cognitive, and social-emotional development in early life and highlighting the need to consider both nutrition and home context when evaluating child development (19, 20). While the developmental risks from undernutrition are well-documented (18, 21–23), there is considerable variability in how children respond to environmental risk. Some children display resilience, developing strong social-emotional competencies despite adversity, whereas others exhibit greater social-emotional difficulties (24). Resilience frameworks have traditionally focused on fixed traits like temperament or genetics (25–28), with limited research on modifiable resilience factors—like early nutrition interventions—that could buffer children against the negative effects of low-quality home environments. Investigating these interactive effects on social-emotional development can help identify modifiable factors that buffer children against adverse conditions in LMICs and can better inform integrated intervention design.

Systematic reviews and meta-analytic evidence support independent and additive effects of adequate nutrition and stimulating home environments in promoting a broad range of child developmental outcomes (e.g., physical, motor, cognitive, language, and social development) for children in LMICs (18, 29–32). Indeed, recent meta-analytic evidence shows that integrated interventions targeting both nutrition and psychosocial stimulation (i.e., interventions targeting sensory intervention that the child receives from social and environmental interactions) have more pronounced language and cognitive benefits for undernourished children as compared to nutrition supplementation only (33). However, it is unclear which specific aspects of integrated interventions are uniquely associated with positive outcomes. Identifying the key elements of effective integrated interventions is crucial for designing high-quality, scalable, and context-specific interventions for children living in LMICs (17, 32, 33).

Small-quantity lipid-based nutrient supplements (LNS) are a recommended preventative strategy for addressing maternal and child undernutrition and optimizing children's health, growth, and development in LMICs (34) with meta-analytic evidence suggesting that small-quantity LNS for children 6–23 months of age reduces the risk for child stunting, wasting, anemia, mortality and developmental delay (35). The International Lipid-Based Nutrient Supplements (iLiNS) DYAD-Ghana trial—a randomized controlled trial of pre- and postnatal small-quantity LNS supplementation—was designed to evaluate the efficacy of LNS in preventing multiple nutritional limitations common in LMICs, such as micronutrient deficiencies, inadequate intake of essential fatty acids (EFAs), and deficiencies in several key macrominerals (36), and in preventing nutrition-related risks (e.g., low birth weight, stunting, wasting, and development delay) (1–3).

By targeting both prenatal and postnatal periods, the LNS intervention was hypothesized to enhance developmental trajectories during the first 1,000 days—a sensitive window for promoting long-term cognitive and socio-emotional outcomes (19, 22, 23). Maternal and infant diets during the first 1,000 days frequently do not provide adequate iron, zinc, vitamin A, and essential fatty acids, especially for women and children living in LMICs; therefore, daily SQ-LNS during this sensitive window is recommended to meet these nutrient requirements and promote optimal growth and developmental outcomes, especially for children in low-resource settings (37). The supplement provided a small daily dose (20 g/day) of energy-dense food (118 kcal/d, 2.6 g protein/d) that included a full complement of micronutrients, and four macrominerals not usually present in multiple micronutrient supplements (calcium, phosphorus, potassium, and magnesium) in combination with EFAs (e.g., α-linolenic acid, a precursor to DHA). Detailed descriptions of the LNS composition have been published elsewhere (36, 38, 39) and are presented in Table 1.

**Table 1:** Nutrient and energy contents of the iron and folic acid capsule, multiple micronutrient supplement capsule, and small-quantity lipid-based nutrient supplement used in the international lipid-based nutrient supplements (iLiNS) DYAD Ghana trial.

**Nutrient**	**IFA**	**MMN**	**Maternal LNS**	**Child LNS**
Ration per day	1 capsule	1 capsule	20-g sachet	20-g sachet
Total energy (kcal)	0	0	118	118
Protein (g)	0	0	2.6	2.6
Fat (g)	0	0	10	9.6
Linoleic acid (g)	0	0	4.59	4.46
α-Linolenic acid (g)	0	0	0.59	0.58
Vitamin A (μg RE)	0	800	800	400
Vitamin C (mg)	0	100	100	30
Vitamin B-1 (mg)	0	2.8	2.8	0.3
Vitamin B-2 (mg)	0	2.8	2.8	0.4
Niacin (mg)	0	36	36	4
Folic acid (μg)	400	400	400	80
Pantothenic acid (mg)	0	7	7	1.8
Vitamin B-6 (mg)	0	3.8	3.8	0.3
Vitamin B-12	0	5.2	5.2	0.5
Vitamin D (mg)	0	10	10	5
Vitamin E (mg)	0	20	20	6
Vitamin K (μg)	0	45	45	30
Iron (mg)	60	20	20	6
Zinc (mg)	0	30	30	8
Copper (mg)	0	4	4	0.34
Calcium (mg)	0	0	280	280
Phosphorus (mg)	0	0	190	190
Potassium (mg)	0	0	200	200
Magnesium (mg)	0	0	65	40
Selenium (μg)	0	130	130	20
Iodine (μg)	0	250	250	90
Manganese (μg)	0	2.6	2.6	1.2

IFA, iron and folic acid capsule; MMN, multiple micronutrient supplement capsule; LNS, small-quantity lipid-based nutrient supplement. Information from table has been previously published elsewhere (38, 39).

In previous work, we found that young Ghanaian children whose mothers were randomized to receive LNS during pregnancy and for the first 6 months postpartum, and who themselves received LNS from 6 to 18 months of age, had significantly lower overall social-emotional difficulties at 4–6 years of age, compared to children in the control group (40). Furthermore, the effect of LNS on social-emotional difficulties was greater among children living in lower quality home environments compared to children in higher quality homes. These findings suggested that early-life nutritional supplementation might be a modifiable resilience factor, protecting Ghanaian children's social-emotional development in lower quality home environments.

Building upon this, the current study had three aims. First, we examined which *specific* social-emotional outcomes were associated with early-life nutritional supplementation to better understand how LNS may influence social-emotional strengths (i.e., prosocial behavior) and social-emotional difficulties (i.e., emotional, conduct, peer relationship, hyperactivity/inattention problems, and total difficulties). Second, we examined which *specific* aspects of the home environment were associated with specific social-emotional strengths and difficulties in this cohort. We built upon our previous work by investigating how to meaningfully characterize the Ghanaian home environment, adapting the subscales of the Early Childhood Home Observation for the Measurement of Environment (EC-HOME) inventory (41, 42) to account for Ghanaian culture and provide a more accurate measure of specific environmental factors relevant to Ghanaian child development. Third, we examined whether early-life nutritional supplementation modified the associations between specific aspects of the home environment and social-emotional strengths and difficulties and the extent to which LNS buffered against social-emotional risk in lower quality home environments.

## Method and materials

### Study design and participants

**Figure 1:**
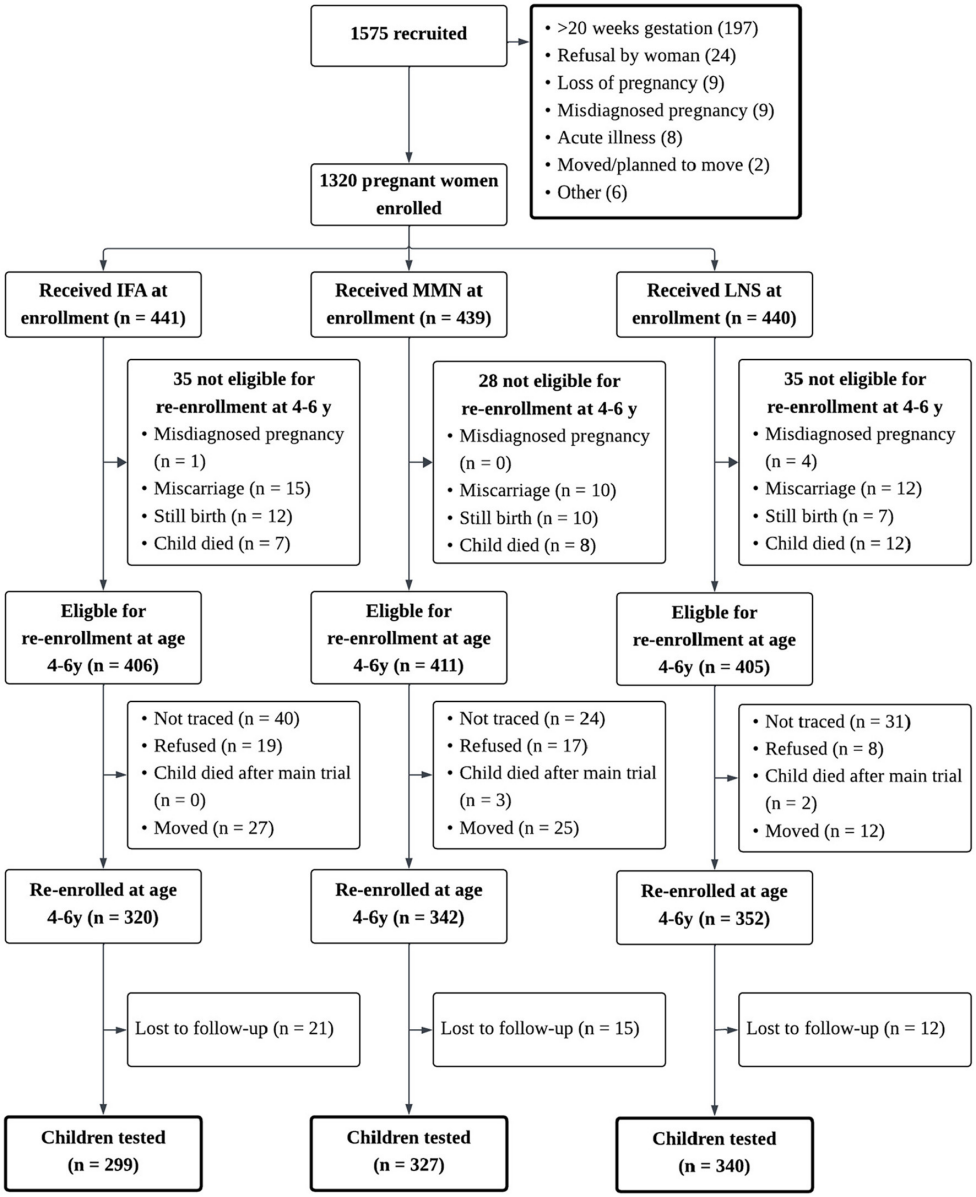
Study profile of the international lipid-based nutrient supplements (iLiNS) DYAD Ghana trial. Study profile showing infants whose mothers were enrolled into the trails, and reasons for participants being lost to follow-up. IFA, iron and folic acid capsule; MMN, multiple micronutrient supplement capsule; LNS, lipid-based nutrient supplement. In the LNS group, women received 20g LNS daily during pregnancy and 6 mo postpartum; infants received 20g LNS daily from 6 to 18mo of age. In the Control group (i.e., IFA or MMN), women received either IFA during pregnancy and placebo for 6 mo postpartum or MMN capsules during pregnancy and 6 mo postpartum. Infants in the Control group did not receive any supplement. Groups shown are based on supplements women received at enrollment.

Between 2009 and 2014, the iLiNS-DYAD-Ghana trial was conducted in semi-urban communities in the Eastern Region of Ghana. A total of 1,320 pregnant women were randomly assigned to 1 of 3 daily intervention arms from enrollment to delivery: (1) 60 mg of iron plus 400 μg of folic acid [iron and folic acid (IFA) group: *n* = 441]; (2) multiple micronutrient capsule containing 18 vitamins and minerals [multiple micronutrients (MMN) group: *n* = 439]; and (3) LNS with similar micronutrients as the MMN supplement, plus other minerals and macronutrients (LNS group: *n* = 440). LNS was a suspension-based colloid and was not microencapsulated. Detailed information about LNS formulation and acceptability in pregnant and lactating Ghanaian women and their infants has been published elsewhere (43, 44). After birth, women in the MMN and LNS groups continued to receive the same supplements until 6 months postpartum, whereas the control IFA group received a calcium placebo capsule (200 mg/d) during that period. Children in the LNS group received LNS designed for children from 6 to 18 months of age, whereas children in the other 2 groups received no supplement (36). Details of the study profile are presented in Figure 1, and detailed information about the nutrient and energy contents of the study's dietary supplements are in Table 1. Maternal and child biological samples (blood, urine, and breastmilk) were collected for biomarker assays, and detailed protocols and results are available elsewhere (45–49). In this analysis, we considered the LNS group as the intervention group and the IFA and MMN groups were combined to be the control group, as previous analyses have not shown significant differences between the IFA and MMN groups in social-emotional outcomes (40).

Between January and December 2016, all parents or caregivers of children who had participated in the iLiNS-DYAD-Ghana trial were contacted for enrollment in the follow-up study. During this period, children who had been born during the trial were 4–6 years of age. We re-enrolled 1,014 children whose mothers or caregivers provided informed consent to participate, and we obtained data from 966 participants (40). Ethical approval for this follow-up study was obtained from the Institutional Review Board of the University of California, Davis, the Ethics Committee for the College of Basic and Applied Sciences of the University of Ghana, and the Ghana Health Service Ethical Review Committee.

### Data collection

Detailed descriptions of the study procedures for the randomized trial and the follow-up study—including randomization procedures and pre-intervention covariate balance checks—have been published elsewhere (36, 40). Information relevant to this secondary analysis is summarized here. At enrollment into the original trial, maternal and household information, including maternal education and household assets, was collected by trained fieldworkers using a questionnaire. In 2016, field staff visited participants' homes to explain the follow-up study, obtain consent for participation, and collect updated sociodemographic information. Consenting participants were scheduled to visit the test center for neurodevelopmental measures. An additional home visit was conducted to administer the EC-HOME to measure various aspects of the home environment.

### Measures

#### EC-HOME inventory

We measured children's home environment using the EC-HOME, which we adapted to assess the quality of children's home environment in the local context. The adapted version of the EC-HOME included 46 items scored on a binary (Yes-No) scale to measure children's home environment. Details on how the EC-HOME was adapted and used to assess the quality of children's home environment in the local context have been previously published (40). Past research with the EC-HOME has used eight subscales that characterize the quality of the home environment based on Learning Materials, Language Stimulation, Physical Environment, Caregiver Responsivity, Academic Stimulation, Desirable Behavior Modeling, Family Lifestyle Variety, and Negative Behavior Acceptance. However, those subscales were developed using samples representative of young children in the United States (41, 42). Considering the cultural and language differences, the same subscales may be inappropriate to measure home environments in Ghana. Therefore, we conducted an exploratory factor analysis (EFA) to investigate the underlying factors of the adapted version of EC-HOME in the local context in Ghana. After performing the EFA, we dropped 14 items, resulting in an adapted scale with 32 items and 5 factors (see Supplementary material S1 for details).

A confirmatory factor analysis showed that the 5-factor model fit our data well [$\chi_{\left(496\right)}^{2}$ = 921.98, CFI = 0.97, RMSEA = 0.04]. The Cronbach's alpha of the five new subscales (ranged from 0.43 to 0.79) and the mean item-total correlations of those subscales (ranged from 0.45 to 0.83) were improved from the Cronbach's alpha of the eight standard subscales (ranged from 0.14 to 0.69) and their mean item-total correlations (ranged from 0.45 to 0.69) originally calculated with our sample (Supplementary Table S2). Collectively, these results suggested that the following 5 subscales meaningfully characterized the Ghanaian home environment: Learning Materials (10 items, “Child has toys or games or posters which help teach letters/alphabet”), Physical Environment (i.e., a more physically safe household free of common building hazards that provides a clean living space; 3 items, “Outside play environment appears safe”), Caregiver Responsivity (8 items, “Parent hugs, kisses, or holds child during visit”), Academic & Language Stimulation (7 items, “Child is encouraged to learn numbers”), and Negative Behavior Acceptance (i.e., avoiding punitive control; parent's ability to accept negative behavior from the child as something to be expected from young children rather than as an act demanding immediate harsh reprisal, 4 items, “Parent does not use physical restraint during visit”). All items for each subscale are presented in Supplementary Tables S1, S2.

#### Strengths and difficulties questionnaire

We assessed child social-emotional competence and difficulties by caregiver interview using the Strengths and Difficulties Questionnaire (SDQ) (50). The SDQ is comprised of 25 items divided into 5 subscales: (1) Emotional Symptoms, (2) Conduct Problems, (3) Hyperactivity/inattention, (4) Peer Relationship Problems, and (5) Prosocial Behavior. Each item is scored on a 3-point scale with 0 = “not true,” 1 = “somewhat true,” and 2 = “certainly true.” We translated the measure into the local languages. Subscale scores can be computed by adding scores on relevant items (after recoding reversed items; range 0–10). Cronbach's alpha for the total difficulties score was 0.62, and for the subscales ranged from 0.66 to 0.77 (see Supplementary Table S2 for further detail).

Clinical cut-off scores for the subscales are: Emotional Symptoms ≥5, Conduct Problems ≥4, Hyperactivity/Inattention ≥7, Peer Relationship Problems ≥4, and Prosocial Behavior ≤ 4. The subscales 1–4 are added to generate a “Total Difficulties” scale (range 0–40) with the clinical cut-off score being ≥17. Importantly, the SDQ is not a clinical diagnostic tool and cannot confirm or rule out a mental health diagnosis. The SDQ has been used as a screening instrument, with clinical cut-off scores indicating elevated and potentially clinically concerning behavior (12, 14). Of note, SDQ clinical cut-off scores have been normed in predominantly high-income countries (51), and there is mixed evidence supporting these clinical cut-off scores as valid for African children (4). For our analyses, we calculated clinical cut-offs for descriptive purposes, to more comprehensively characterize children in the LNS and control groups. Raw scale scores were used for hypothesis testing.

#### Covariates

We controlled for data collector, child age, gender, years of maternal education, maternal age, household assets index, maternal depression, and maternal agency in hypothesis testing analyses. All maternal covariates were measured at baseline. Detailed descriptions of the creation of these variables have been published elsewhere (36, 40), and brief descriptions of questionnaire-based covariates are provided here.

##### Home assets index

We created a proxy indicator for household socioeconomic status for each household based on ownership of assets (e.g., radio, television), lighting sourcing, drinking water supply, sanitation facilities, and flooring materials. Using principal components analysis, household ownership of these assets was combined into an index with a mean of zero and standard deviation of 1. Higher values represent higher socioeconomic status.

##### Maternal depression

We used the 10-item Edinburg Postnatal Depression Scale (EPDS) to measure maternal depressive symptoms at baseline, which has been validated among both postpartum and non-postpartum women (52). Mothers rated items on how frequently they had experienced the described symptom in the past week (e.g., “I have felt sad or miserable”). Mothers rated each item on a 4-point scale, and 7 items were reverse-scored. Items were summed together, and total scores could range from 0 to 30. Higher EPDS scores indicate more depressive symptoms, with scores ≥12 suggesting severe depressive symptoms.

##### Maternal agency

We used the 10-item General Self-Efficacy Scale (GSES) to measure maternal agency and general sense of perceived self-efficacy at baseline (53). Mothers rated how well an item described them (e.g., “I can usually handle whatever comes my way”) on a 4-point scale. Items were summed together with higher GSES scores reflecting more maternal agency and self-efficacy (range 10 to 40).

### Statistical analysis

#### Study pre-registration

We posted a statistical analysis plan with pre-specified potential covariates to the OSF website (https://osf.io/bmv9d/). Analyses were performed using R version 4.4.1. The R package “lavaan” (54) was used to run multi-group comparison of the path analyses. Results with a *p*-value < 0.05 were considered significant, and results with a *p*-values of < 0.10 were considered marginally significant.

#### Attrition and missing data

Of the 1,320 women enrolled in the trial, 966 children were assessed in the follow-up study. Only four children had completely missing EC-HOME and SDQ measures; they were excluded from hypothesis testing analyses. Children included in this analysis (*n* = 962) did not differ from those excluded (*n* = 358) in background characteristics such as child sex, maternal education, household asset scores, maternal depression, and maternal agency (*p*'s > 0.13). However, a greater proportion of children included in the analysis were in the LNS group (35.1%) compared to LNS representation in those excluded from analyses (28.7%; *p* = 0.03), which indicates more children in the control group were lost to follow up compared to the LNS group (Supplementary Table S3). Little's Missing Completely at Random test suggested that data could be treated as missing completely at random, $\chi_{\left(247\right)}^{2}$ = 269.97, *p* = 0.15.

#### Hypothesis testing

**Figure 2:**
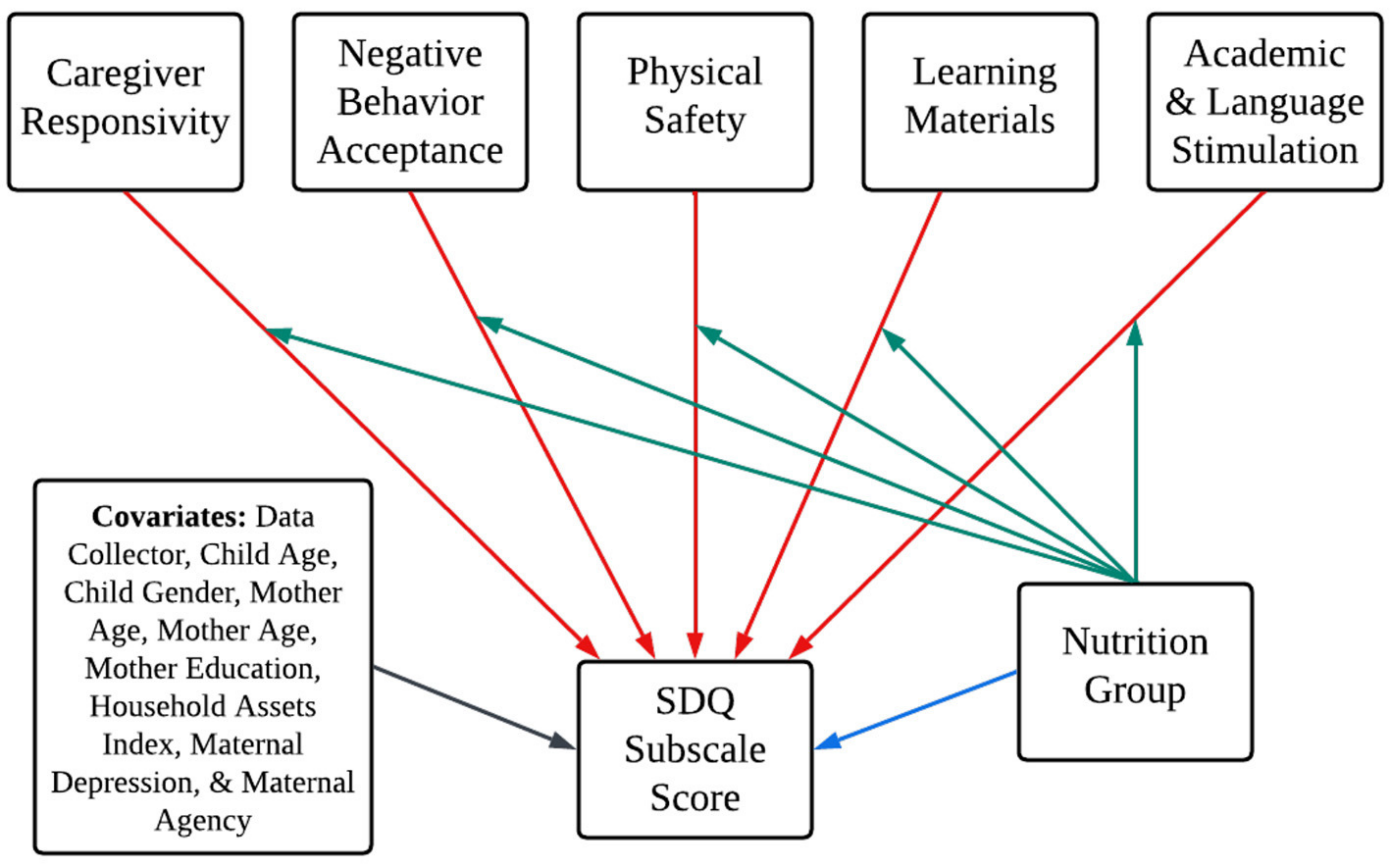
Conceptual figure of model paths illustrating the study aims. Conceptual figure of path models illustrating the study aims. The blue path corresponds to Aim 1, the red paths correspond to Aim 2, and green paths correspond to Aim 3. Covariances removed to aid in model clarity. Nutrition Group was dummy-coded (1 = LNS, 0 = Control). Six models fit for each of the six SDQ subscale scores. The blue path was removed for multiple group comparison testing for Aim 3.

As part of preliminary data screening, we assessed univariate normality for all continuous variables by examining skewness and kurtosis values. All variables fell within acceptable thresholds for univariate normality, defined as skew values within ±2 and kurtosis values within ±7 (55). To test Aims 1 and 2, we fit six path models regressing the EC-HOME subscales, nutrition group (dummy-coded, 1 = LNS, 0 = Control), and covariates onto each SDQ Subscale Score: Total Difficulties, Emotional Problems, Conduct Problems, Peer Relationship Problems, Hyperactivity/Inattention, and Prosocial Behavior. Figure 2 provides a conceptual figure of these path models. Variables with significant zero-order correlations were covaried in each path model. For Aim 3, we used multiple group comparison to examine the potential moderating effect of early-life nutritional supplementation (LNS vs. control group) on the association of each EC-HOME subscale with each of the six continuous SDQ subscale scores. First, we fit a model removing nutrition group as a predictor and then freely estimating all parameters for both nutrition groups. Next, a second model was fit constraining all paths between the EC-HOME subscales and the SDQ subscale to be equal across nutrition groups, and a chi-square goodness of fit was performed to determine whether the constrained model significantly worsened model fit. If the unconstrained model was supported, the Wald test with cross-group equality constraints for each regression path was used to test for treatment group difference in path coefficients.

For all models, we assessed model fit with the chi-square likelihood ratio statistic and these fit index criteria: RMSEA < 0.08, SRMR < 0.08, CFI > 0.90, and TLI > 0.90 (56). It should be noted that SEM analyses with large sample sizes yield the smallest confidence intervals around parameter estimates; subsequently, the likelihood-ratio test becomes overpowered and more likely to reject models due to small differences in residuals despite the tested model being well-specified (57). Hence, the RMSEA, SRMR, CFI, and TLI indices are more discerning for model fit assessment with our sample size. Full information maximum likelihood (FIML) was used to estimate missing data values. We conducted *post hoc* power analyses and report this information in the Supplementary Table S6.

## Results

### Preliminary analyses

Table 2 shows the participants' demographic characteristics, EC-HOME subscale raw scores, and the raw scores of the SDQ subscales in the LNS and the control groups. Independent samples *t-*tests found that participants in the LNS group were from households with a lower mean asset score than the control group (*p* = 0.02). There were no other significant differences between the two groups (*p*'s ≥ 0.05). Of note, group differences were approaching significance for severe maternal depressive symptoms (total scores ≥ 12), SDQ Peer Relationship Problems, and Total Difficulties. Mothers in the LNS group had a marginally greater prevalence of severe depressive symptoms at baseline, with 25.1% of mothers in the LNS group reporting severe depressive symptoms and 20.4% of mothers in the control group reporting severe depressive symptoms [χ^2^_(1)_ = 2.80, *p* = 0.09]. Overall, these findings suggest that the nutritional groups did not substantively differ on background characteristics, despite attrition. For the SDQ subscales, the control group had marginally higher Peer Relationship Problems (*p* = 0.05) and Total Difficulties scores (*p* = 0.07).

**Table 2:** Comparison of participant characteristics of the LNS group vs. the control group.

**Variable**	**LNS Mean ±SD [n] or % [n/total] *n* = 338**	**Control Mean ±SD [n] or % [n/total] *n* = 624**	***t*(df)**	***p*-value**
Child Age (y)	4.96 ± 0.56	4.90 ± 0.55	−1.68(960)	0.09
Male (%)	48.52% [164/338]	47.44% [296/624]	−0.30(959)	0.77
Mother Age (y)	26.88 ± 5.48	26.76 ± 5.50	−0.32(960)	0.75
Maternal Education (y)^A^	7.66 ± 3.72	7.58 ± 3.41	−0.34(960)	0.74
Maternal Depression^B^	6.08 ± 5.79	5.56 ± 5.68	−1.32(948)	0.19
Maternal Agency^C^	25.06 ± 5.07	24.79 ± 4.77	−0.78(913)	0.43
**Household Asset Index**	**−0.09** **±0.96**	**0.06** **±0.96**	**2.28(957)**	**0.02** ^*^
**EC-HOME subscales**				
Caregiver Responsivity	6.37 ± 1.25	6.35 ± 1.35	−0.22(821)	0.83
Negative Behavior Acceptance	3.24 ± 0.85	3.17 ± 0.92	−1.12(844)	0.26
Learning Materials	2.91 ± 1.97	2.97 ± 2.12	0.42(925)	0.67
Physical Environment	2.60 ± 0.84	2.56 ± 0.88	−0.64(844)	0.52
Academic & Language Stimulation	5.89 ± 1.22	5.85 ± 1.27	−0.44(926)	0.66
**SDQ subscales**				
Emotional Problems	3.00 ± 1.97	3.10 ± 2.05	0.71(956)	0.48
Conduct Problems	2.64 ± 1.58	2.82 ± 1.75	1.57(956)	0.12
Hyperactivity & Inattention	5.24 ± 1.76	5.28 ± 1.80	0.36(956)	0.72
Peer Relationship Problems	2.24 ± 1.45	2.44 ± 1.56	1.93(956)	0.05
Prosocial Behaviors	7.88 ± 1.82	7.88 ± 1.93	0.06(956)	0.95
Total Difficulties	13.13 ± 4.13	13.65 ± 4.55	1.79(745.30)^D^	0.07

Significant group differences are in bold. ^A^In Ghana, the average number of years of schooling for women is around 11.5 years; however, this varies significantly based on socioeconomic status and location (112). ^B^Maternal Depression scores range from 0 to 30 with scores ≥12 indicating severe depressive symptoms (52). ^C^Maternal Agency scores range from 10 to 40 with higher scores reflecting more agency. ^D^Welch's *t-*test reported as homogeneity of variance was violated. ^*^ *p* < 0.05.

Table 3 documents the prevalence of children's social-emotional difficulties above the SDQ clinical cut-offs on the four SDQ problem subscales, the prevalence of low scores on the prosocial scale, and the chi-square test for equality of proportions to test for significant differences in prevalence between the two groups. The LNS group had significantly lower prevalences of conduct problems and total difficulties scores in the clinically concerning range compared to the control group. Finally, we performed zero-order correlations between participants' demographic characteristics, EC-HOME subscale raw scores, and SDQ subscale raw score for the entire sample to identify covariances to include for hypothesis testing. Supplementary material contains a complete description of zero-order correlations (Supplementary Table S4).

**Table 3:** Prevalence of children with clinical cut-off scores for social-emotional strengths and difficulties.

**Subscales**	**Clinical cut-offs**	**Prevalence (Full sample)**	**Prevalence (LNS)**	**Prevalence (Control)**	***p*-value of chi-square**
		*n =* 962	*n* = 338	*n* = 624	
Emotional problems	≥5	23.6%	20.8%	25.8%	0.14
**Conduct problems**	≥**4**	**33.1%**	**28.9%**	**35.4%**	**0.04** ^*^
Hyperactivity & inattention	≥7	26.5%	26.2%	26.7%	0.87
Peer problems	≥4	22.2%	18.8%	24.1%	0.06
Prosocial behaviors	≤ 4	3.6%	3.6%	3.5%	0.98
**Total difficulties**	≥**17**	**25.3%**	**21.1%**	**27.5%**	**0.02** ^*^

Clinical cut-off scores validated based on children from predominantly Western countries. Significant group differences are in bold. ^*^ *p* < 0.05.

### Aims 1 and 2: examine how the home environment and early-life nutritional supplementation predict social-emotional outcomes

We fit six path models to examine how the five EC-HOME factors (Aim 1) and nutritional supplementation (Aim 2) predict children's SDQ scores (emotional problems, conduct problems, hyperactivity/inattention, peer relationship problems, total difficulties, and prosocial behavior; see Table 4). We fit all six path models with the covariances shown in Supplementary Figure S2, which reflect the significant correlations between participant demographics (Supplementary Table S4). Model fit and unstandardized path estimates for EC-HOME factors predicting SDQ scores for all six models are presented in Table 4. Complete model parameter estimates for all six models are presented in Supplementary Table S5. Holistically, the comparative and absolute fit indices for all six models indicate that our models had good fit despite the significant likelihood-ratio test.

**Table 4:** Unstandardized path estimates for early-life nutritional supplementation and home environment factors predictive of Ghanaian children's social-emotional strengths & difficulties.

	**Model 1: emotional problems**	**Model 2: conduct problems**	**Model 3: hyperactivity & inattention**	**Model 4: peer relationship problems**	**Model 5: total difficulties**	**Model 6: prosocial behavior**
**Predictor**	***b*** **(SE)**	***b*** **(SE)**	***b*** **(SE)**	***b*** **(SE)**	***b*** **(SE)**	***b*** **(SE)**
Nutritional supplement group^A^	−0.12 (0.12)	**−0.20 (0.11)** ^†^	−0.06 (0.12)	**−0.22 (0.10)** ^*^	**−0.58 (0.26)** ^*^	0.01 (0.12)
Caregiver responsivity	−0.03 (0.05)	**−0.12 (0.04)** ^**^	**−0.10 (0.05)** ^*^	−0.03 (0.04)	**−0.29 (0.11)** ^**^	**0.20 (0.05)** ^***^
Negative behavior acceptance	–0.26 (0.07)^***^	**−0.23 (0.06)** ^***^	**−0.22 (0.07)** ^**^	**−0.27 (0.06)** ^***^	**−0.99 (0.15)** ^***^	−0.07 (0.07)
Learning materials	−0.05 (0.03)	**−0.06 (0.03)** ^*^	−0.04 (0.03)	0.01 (0.03)	**−0.14 (0.07)** ^*^	**0.07 (0.03)** ^*^
Physical safety	−0.12 (0.08)	−0.02 (0.07)	−0.01 (0.07)	0.01 (0.06)	−0.12 (0.16)	0.05 (0.08)
Academic & language stimulation	**0.14 (0.05)** ^**^	−0.02 (0.04)	**−0.11 (0.05)** ^*^	−0.02 (0.04)	−0.01 (0.11)	0.02 (0.05)
Maternal depression	**0.12 (0.01)** ^***^	**0.06 (0.01)** ^***^	**0.04 (0.01)** ^***^	**0.02 (0.01)** ^*^	**0.24 (0.02)** ^***^	**−0.022 (0.011)** ^*^
Maternal agency	**−0.03 (0.01)** ^*^	**−0.03 (0.01)** ^**^	**−0.03 (0.01)** ^*^	**−0.02 (0.01)** ^†^	**−0.11 (0.03)** ^***^	**0.031 (0.013)** ^*^
Child age	**−0.49 (0.11)** ^***^	**−0.25 (0.10)** ^*^	−0.01 (0.11)	**−0.23 (0.09)** ^*^	**−0.98 (0.24)** ^***^	**0.348 (0.37)** ^**^
Child gender^B^	**−0.24 (0.12)** ^*^	0.12 (0.10)	**0.22 (0.11)** ^*^	−0.06 (0.09)	−0.04 (0.25)	**−0.294 (0.116)** ^*^
Mother age	−0.01 (0.01)	0.01 (0.01)	**−0.04 (0.01)** ^***^	−0.01 (0.01)	**−0.05 (0.02)** ^*^	**0.0219 (0.011)** ^*^
Years of maternal education	−0.01 (0.02)	0.01 (0.02)	0.01 (0.02)	0.01 (0.01)	0.01 (0.04)	0.011 (0.018)
Household assets index	−0.09 (0.07)	−0.03 (0.06)	−0.06 (0.06)	−0.06 (0.05)	**−0.24 (0.14)** ^†^	0.038 (0.067)
Field Staff 1	−0.23 (0.19)	**−0.44 (0.16)** ^**^	0.21 (0.18)	−0.10 (0.15)	−0.55 (0.39)	**−0.350 (0.183)** ^†^
Field Staff 2	**−0.31 (0.18)** ^†^	**−0.56 (0.16)** ^**^	−0.05 (0.17)	−0.11 (0.15)	–1.03 (0.39)^**^	**0.526 (0.181)** ^**^
Field Staff 3	−0.28 (0.21)	−0.30 (0.18)	−0.09 (0.20)	**−1.08 (0.17)** ^***^	–1.73 (0.44)^***^	**0.509 (0.209)** ^*^
Field Staff 4	−0.04 (0.19)	**−0.50 (0.16)** ^**^	0.10 (0.17)	**−0.27 (0.15)** ^†^	−0.29 (0.39)	0.055 (0.183)
Model Fit:	χ^2^_(78)_ = 102.16, *p* = 0.04, CFI = 0.99, TLI = 0.97, RMSEA = 0.02, SRMR = 0.03	χ^2^_(78)_ = 102.58, *p* = 0.03, CFI = 0.99, TLI = 0.97, RMSEA = 0.02, SRMR = 0.03	χ^2^_(78)_ = 103.22, *p* = 0.03, CFI = 0.99, TLI = 0.97, RMSEA = 0.02, SRMR = 0.03	χ^2^_(78)_ = 103.58, *p* = 0.03, CFI = 0.99, TLI = 0.97, RMSEA = 0.02, SRMR = 0.03	χ^2^_(66)_ = 87.52, *p* = 0.04, CFI = 0.99, TLI = 0.98, RMSEA = 0.02, SRMR = 0.03	χ^2^_(78)_ = 102.51, *p* = 0.03, CFI = 0.99, TLI = 0.97, RMSEA = 0.02, SRMR = 0.03

*N* = 962. ^A^Nutritional Supplement Group was dummy coded (1 = LNS Group, 0 = Control Group). ^B^Child Gender was dummy-coded (1 = Male, 0 = Female). Field Staff distinguishes data collectors. Significant paths are in bold. Covariance estimates not presented to aid with interpretability.^†^*p* < 0.10, ^*^ *p* < 0.05, ^**^ *p* < 0.01, ^***^ *p* < 0.001.

For Aim 1, we found that children who received LNS had fewer total difficulties (β = −0.064, *p* = 0.023), fewer peer relationship problems (β = −0.07, *p* = 0.03), and tended to have fewer conduct problems (β = −0.06, *p* = 0.07) at age 4–6 years. These associations are aligned with our previous work that found children who received LNS had significantly lower total difficulties' z-scores compared to children in the control group (40). Nutritional supplementation was not significantly associated with children's emotional problems and hyperactivity/inattention, nor was it associated with children's prosocial behavior (*p*'s > 0.32).

In support of Aim 2, distinct and specific aspects of the home environment were uniquely associated with children's SDQ scores at age 4–6, particularly in relation to social-emotional difficulties. More responsive caregiving was associated with fewer conduct problems (β = −0.10, *p* = 0.001), less hyperactivity/inattention (β = −0.09, *p* = 0.017), fewer total difficulties (β = −0.09, *p* = 0.003), and greater prosocial behavior (β = 0.14, *p* < 0.001). Caregivers who were more accepting of their children's age-appropriate negative behavior (i.e., used less punitive discipline) had children with fewer emotional (β = −0.12 *p* < 0.001), conduct (β = −0.12, *p* = 0.001), hyperactivity/inattention (β = −0.11, *p* < 0.001), and peer relationship problems (β = −0.163, *p* < 0.001) and fewer total difficulties (β = −0.205, *p* = 0.001). Children with more learning materials also had fewer conduct problems (β = −0.06, *p* = 0.04), fewer total difficulties (β = −0.07, *p* = 0.04), and greater prosocial behavior (β = 0.08, *p* = 0.03). Children with greater academic and language stimulation had less hyperactivity/inattention (β = −0.08, *p* =0.03), but contrary to our expectations, more emotional problems (β = 0.09, *p* = 0.002).

Several of the demographic covariates also were significantly associated with children's SDQ scores. For example, mothers who reported more depression or less agency also reported more difficulties and less prosocial behavior for their children. Older children had better adjustment than younger children. Girls had more emotional problems and prosocial behavior than boys, but fewer hyperactivity/inattention problems.

### Aim 3: examine if nutritional supplementation modifies the associations between home environment and children's social-emotional outcomes

Multi-group comparison analysis showed that nutritional supplementation moderated the associations between the home environment and children's hyperactivity/inattention and total difficulties at age 4–6 years (Table 5). Allowing the EC-HOME paths to vary between nutritional supplementation group significantly improved model fit relative to constraining EC-HOME paths to be equal for both groups when predicting children's hyperactivity/inattention [χ^2^_(5)_ = 15.71, *p* = 0.01] and when predicting children's total difficulties [χ^2^_(5)_ = 13.26, *p* = 0.02]. The chi-square goodness of fit tests were non-significant for all other models (*p*'s > 0.40).

**Table 5:** Chi-square goodness of fit tests for multi-group comparison of all models.

**Model**	**Δχ^2^**	**Δdf**	***p*-value**
Model 1: Emotional problems	4.69	5	0.45
Model 2: Conduct problems	4.08	5	0.54
Model 3: Hyperactivity/inattention	**15.71**	**5**	**0.01** ^*^
Model 4: Peer relationship problems	5.14	5	0.40
Model 5: Total difficulties	**13.26**	**5**	**0.02** ^*^
Model 6: Prosocial behavior	4.68	5	0.46

*N* = 962. All tests performed compared an unconstrained model (all paths freely estimated) to a constrained model (all EC-HOME regression paths constrained to be equal across nutritional supplementation groups). Bolded values indicate that the constrained model significantly worsened model fit. ^*^ *p* < 0.05.

#### Nutrition group moderates the associations between the physical environment, academic stimulation, and children's hyperactivity/inattention

**Figure 3:**
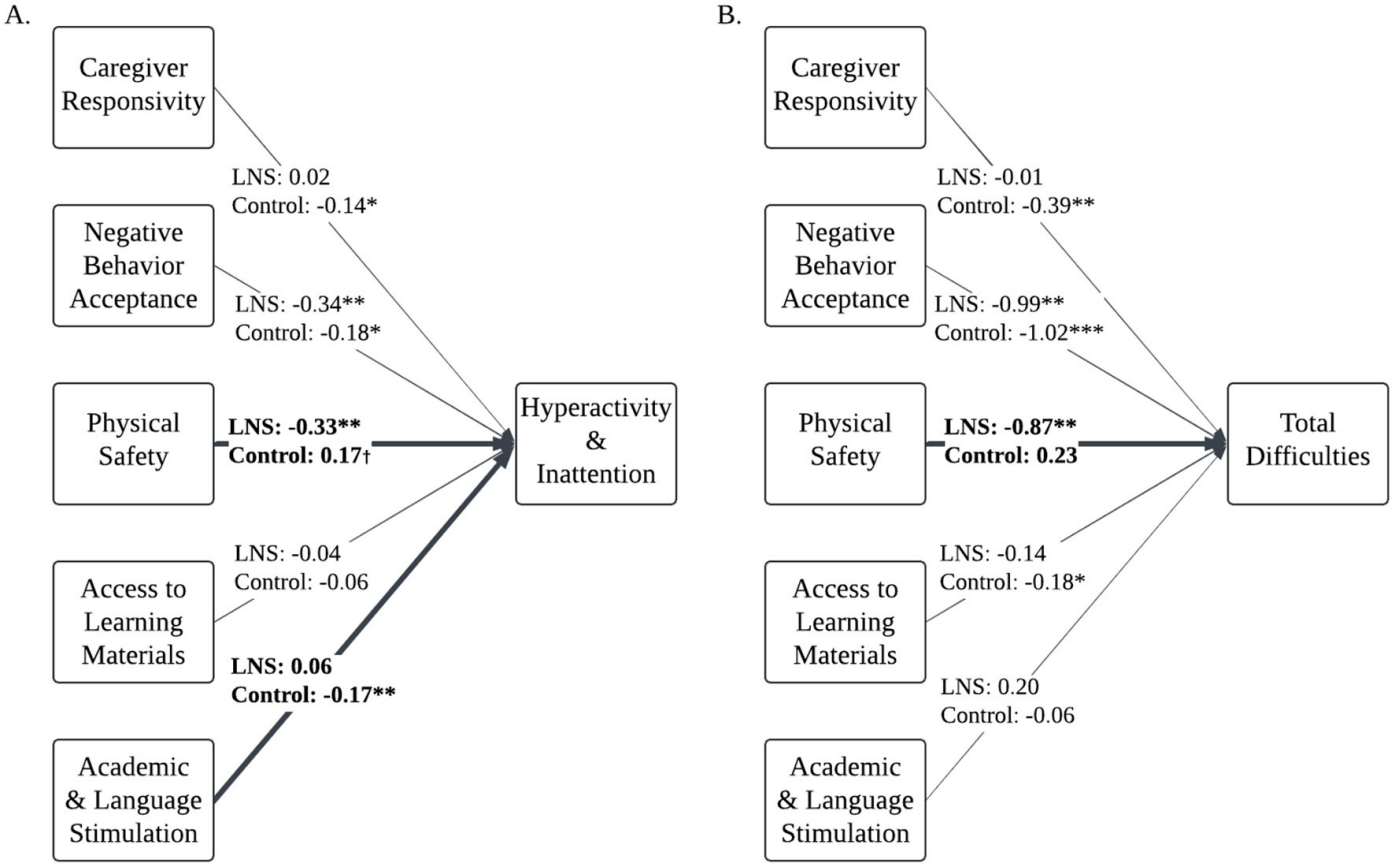
Multi-group comparison path models: associations between home environment and children's hyperactivity/inattention and total difficulties are moderated by nutritional supplementation. *N* = 962. Path models of associations between home environment factors and Ghanaian children's hyperactivity/inattention **(A)** and total difficulties **(B)**, controlling for child age, child gender, mother age, maternal education, household assets index, and data collectors. Multi-group comparison found that the associations between home environment factors and total difficulties and hyperactivity/inattention vary by nutritional supplement group. Bolded, solid lines reflect paths that were significantly different between groups. Values are unstandardized regression estimates. Covariates and covariances not presented to aid with interpretability. Missing data is estimated with Full Informaiton Maximum Likelihood.^†^*p* < 0.10, * *p* < 0.05, ** *p* < 0.01, *** *p* < 0.001.

**Figure 4:**
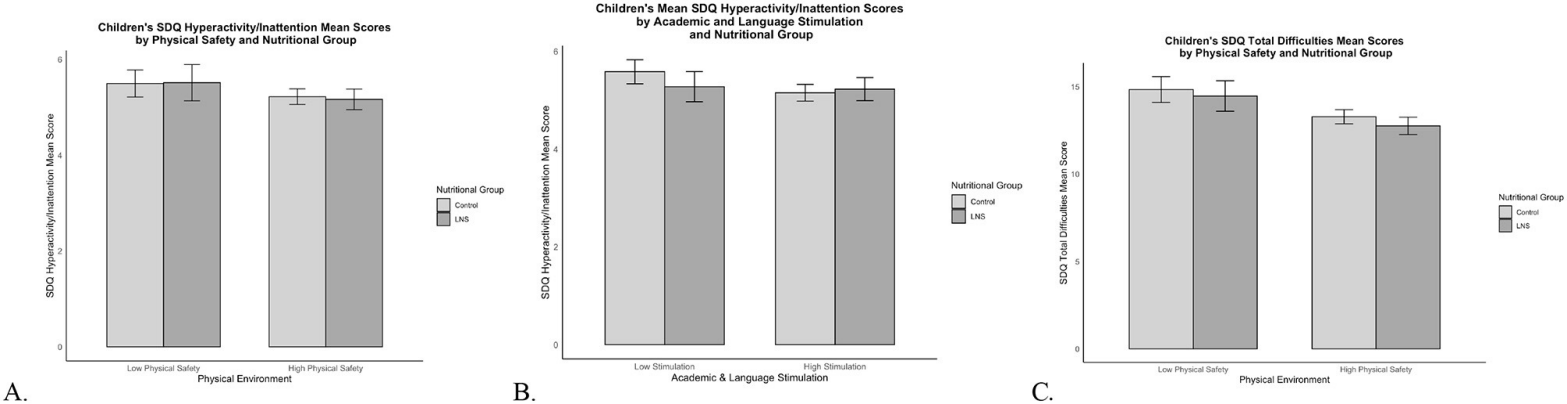
SDQ hyperactivity/inattention and total difficulties mean scores by nutritional supplementation group and HOME subscale scores. Mean SDQ scores by nutritional supplementation group and HOME subscale scores. Error bars show the 95% confidence interval around the mean. **(A)** SDQ hyperactivity/inattention mean scores for LNS and control children living in Low Physical Safety (scores < 3) and High Physical Safety (scores ≥3) homes. **(B)** SDQ hyperactivity/inattention mean scores for LNS and control children living in Low Stimulation (scores < 6) and High Stimulation (scores ≥6) homes. **(C)** SDQ total difficulties mean scores for LNS and control children living in Low Physical Safety (scores < 3) and High Physical Safety (scores ≥3) homes.

The Wald test with cross-group equality constraints for each EC-HOME regression path demonstrated significant group differences for the physical environment [Δχ^2^_(1)_ = 10.73, *p* = 0.001] and for academic and language stimulation path coefficients [Δχ^2^_(1)_ = 4.95, *p* = 0.03] predicting children's hyperactivity/inattention. Living in a safer physical environment was associated with less hyperactivity/inattention among children who received LNS (β = −0.16, *p* = 0.009), whereas the physical environment was not associated with hyperactivity/inattention for the control group (β = 0.08, *p* = 0.06; Figure 3A). To assist with understanding the nature of the moderating influence of physical safety on the predictive associations of nutrition group with hyperactivity/inattention, Figure 4A displays the mean SDQ hyperactivity/inattention scores for children in the LNS vs. control groups, living in the context of less vs. more physically safe homes. Children in the LNS group who lived in physically safer homes had the fewest hyperactivity/inattention problems.

Additionally, less academic stimulation was associated with more hyperactivity/inattention among children in the control group (β = −0.12, *p* = 0.004); but this association was non-significant for children who received LNS (β = 0.04, *p* = 0.46; Figure 3A). To assist with understanding the nature of the moderating influence of academic and language stimulation on the predictive associations of nutrition group with children's hyperactivity/inattention, Figure 4B displays the mean SDQ hyperactivity/inattention scores for children in the LNS vs. control groups, living in the context of less vs. more academically stimulating homes. Children in the control group who experienced less academic and language stimulation had the most hyperactivity/inattention problems. The Wald test was non-significant for all other EC-HOME variables (*p*'s > 0.15), suggesting that those path weights did not significantly differ between nutritional supplementation groups.

#### Nutrition group moderates the associations between the physical environment and children's total difficulties

The Wald test with cross-group equality constraints for each EC-HOME regression path showed significant group differences for the physical environment predicting children's total difficulties [Δχ^2^_(1)_ = 10.64, *p* = 0.001]. Living in a safer physical environment was associated with fewer total difficulties among children who received LNS (β = −0.18, *p* = 0.001), whereas the physical environment was not associated with total difficulties for the control group (β = 0.04, *p* = 0.25; Figure 3B). To assist with understanding the nature of the moderating influence of physical safety on the predictive associations of nutrition group with SDQ problem scores, Figure 4C displays the mean SDQ total problem scores for children in the LNS vs. control groups, living in the context of less vs. more physically safe homes. Children in the LNS group who lived in physically safer homes had the fewest total difficulties. The Wald test was non-significant for all other EC-HOME variables (*p*'s > 0.09), suggesting that those path weights did not significantly differ between nutritional supplementation groups.

## Discussion

Children in LMICs face compounded risks, including undernutrition and environmental adversity, both of which elevate the risk for social-emotional difficulties (1, 3, 9). This study examined the main and interactive effects of early-life nutritional supplementation and specific aspects of home environment quality on Ghanaian children's social-emotional functioning. Overall, our findings indicate that early-life nutritional supplementation with small-quantity lipid-based nutrient supplements and multiple aspects of supportive home environments each have a protective effect on social-emotional outcomes, underscoring the importance of both for reducing social-emotional difficulties. Independent associations of LNS and home environment quality were stronger and more prevalent than interactive effects. Yet there also was evidence that early-life LNS enhanced the degree to which growing up in physically safe homes was associated with better psychological adjustment and protected Ghanaian children from hyperactivity/inattention problems associated with experiencing less academic and language stimulation. Hence, our study builds upon previous research by identifying how early-life nutritional supplementation and *specific* aspects of the home environment relate to *specific* social-emotional outcomes.

### Early-life nutritional supplementation promotes distinct aspects of Ghanaian children's social-emotional functioning

We found that children who received LNS exhibited fewer peer relationship problems and total difficulties, and tended to have fewer conduct problems, while controlling for home quality and background characteristics. Additionally, children who received LNS had a lower prevalence of clinically concerning conduct problems and total social-emotional difficulties, compared to the children in the control group. These findings underscore the potential for nutritional supplementation to reduce social-emotional difficulties, possibly by supporting brain and emotional development through sustained nutrient availability during sensitive developmental periods (19, 22, 58, 59). As posited by Ocansey and colleagues (40), better social-emotional functioning for children in the LNS group may stem from the essential fatty acids and iron present in the supplement that they consumed from ages 6 to 18 months. These nutrients are crucial for neurodevelopmental processes—such as synaptogenesis, myelination, and dopaminergic signaling—that support emotion regulation and executive functioning (19, 60, 61).

The LNS supplement contained 0.5 g of α-linolenic acid (ALA)—a precursor to docosahexaenoic acid (DHA)—and 4.6 g of linoleic acid, and we previously found that mothers who received LNS in our study had significantly higher breastmilk ALA at 6 months postpartum compared to mothers in the IFA and MMN conditions (49). Both animal and human research has found DHA deficiency to negatively impact brain regions related to emotion regulation, with subsequent adverse impacts on learning, behavioral, and emotional outcomes (61–64).

Additionally, iron deficiencies during the 6- to 24-month postnatal period can detrimentally alter structural and functional brain development (e.g., dopaminergic pathways, myelination, dendritic growth) (65), contributing to increased risk for social, emotional, and attentional problems in young children (60, 66–69). It is interesting that LNS supplementation was not associated specifically with children's hyperactivity/inattention as DHA and iron deficiency have been consistently associated with both concurrent and prospective attentional and hyperactivity problems measured years later (64, 65, 68, 69); however, many of these studies measured attentional and hyperactivity problems exclusively without considering other social-emotional and behavioral comorbidities. Further research is needed to understand the biological mechanisms underlying how early-life LNS supplementation relates to some—but not all—social-emotional outcomes.

Other possible biological mechanisms underlying how LNS may influence children's social-emotional development include changes to the gut-brain axis or epigenetic changes. Emerging research on the gut–brain axis suggests that, in the first 1,000 days of life, shifts in the intestinal microbiota feed back to the central nervous system via bi-directional neural, endocrine, and immune routes, altering the synthesis and turnover of key neurotransmitters (70), which may subsequently influence pathophysiological risk for social-emotional difficulties and related mental health disorders (71). Alternatively, growing evidence suggests that early-life adequacy of folic acid and B vitamin micronutrients—which were present in the LNS supplement—may influence DNA methylation and gene expression with downstream influence on mental health across the lifespan (72). Elucidating these pathways will require future studies that integrate nutritional interventions with microbiome, epigenomic, and neuroimaging measures.

### Distinct aspects of the home environment are uniquely related to Ghanaian children's social-emotional functioning

Overall, we found that multiple aspects of a higher-quality home environment—particularly caregiver's behavior—were associated with better social-emotional functioning across a range of outcomes. Caregivers who showed more emotional warmth and verbal responsivity toward their children had children with fewer problems with conduct, hyperactivity/inattention, and overall social-emotional functioning. Additionally, their children showed greater prosociality, a developmental asset associated with academic, physical, and mental wellbeing learning (10, 11). Highly responsive Ghanaian parents model empathy, positive communication, and emotional support for their children and may have more positive parent-child relationships, all of which may help children develop effective emotion regulation and social skills underlying social-emotional competence (73, 74). Ghanaian cultural norms place a strong emphasis on communal interdependence and respect for elders (75); thus, caregiver responsivity may also model and reinforce cooperative social norms and thereby promote children's social-emotional development. These findings are consistent with large-scale, cross-cultural parenting research (76) as well as burgeoning Ghanaian socialization research (77, 78) that suggest caregiver responsivity and warmth to be a “universally beneficial” parenting practice that facilitates children's social-emotional competence.

Similarly, caregivers who demonstrated greater acceptance of children's age-appropriate expressions of negative affect and behaviors and who used less punitive discipline in response to children's negative behavior had children with fewer emotional problems, conduct problems, hyperactivity/inattention, peer relationship problems, and total social-emotional difficulties. Almost all the Negative Behavior Acceptance items described caregivers' use of corporal punishment in response to children's misbehavior (e.g., “*parents neither slaps nor spanks child during visit”*). There is robust meta-analytic and cross-cultural parenting research demonstrating that corporal punishment relates to children's increased short- and long-term risk for behavioral, social, and emotional problems (79, 80), and our findings suggest that Ghanaian parents who refrain from disciplining their children with corporal punishment are also mitigating their children's social-emotional risk. It is possible that, in households with high Negative Behavior Acceptance scores, those parents are using low-power disciplinary strategies, such as inductive reasoning, explaining behavioral expectations, and consistent enforcement of clear rules. These low-power disciplinary strategies provide children with an understanding of the consequences of their behavior and the rationale for why rules are necessary, helping them to internalize social and behavioral norms (81). Indeed, Ghanian parents' greater use of low-power discipline has been related to their children being less aggressive, less depressed, less anxious, and having less peer conflict (77, 78). Nutritional interventions that incorporate parent training should target parents' disciplinary practices.

Regarding the educational climate of the household, children's greater access to learning materials was associated with more favorable social-emotional outcomes whereas the associations of academic and language stimulation within the home to these outcomes were mixed. Children with greater access to learning materials in the home had fewer conduct problems, fewer total difficulties, and greater prosocial behavior, suggesting that educational resources in the household may promote children's positive social-emotional development. Access to learning materials may reduce children's conduct problems and bolster prosocial behaviors by fostering cognitive stimulation, providing fun and interactive activities to practice self-regulation, and promoting positive parent–child interactions through shared activities (e.g., puzzles, story books).

Surprisingly, higher academic and language stimulation was associated with both increased emotional problems and decreased hyperactivity/inattention, suggesting that while academically stimulating home environments may support children's attentional skills, they might also add stress affecting children's emotion regulation. This finding is somewhat inconsistent with research conducted in high-income countries (82) where academically stimulating home environments appear to promote young children's self-regulation (83–85) and cooperative, positive peer interactions (86, 87), while also being associated with children's reduced hyperactivity and problem behaviors (84, 88). Research conducted in high-, middle-, and low-income countries suggests that parental academic pressure contributes to children's heightened stress, anxiety, and mental health risk (89, 90). Parents' excessive academic expectations may undermine children's self-esteem, leading them to internalize feelings of failure or shame if they fail to meet parental standards. Alternatively, children with heightened emotional problems may elicit more academic support from their parents. Future research should aim to disentangle the direction of the association.

Our findings align with previous research that emphasizes the protective role of supportive caregiver behaviors in fostering social-emotional competence (91–94) and the effectiveness of nutritional interventions when paired with stimulating households (30–32, 95). Meta-analytic evidence emphasizes the cumulative, additive benefits of integrating *both* nutrition and responsive caregiving to promote children's social-emotional, cognitive, and physical development (95, 96). That is, both adequate early-life nutrition and high-quality home environments facilitate children's wellbeing, and interventions that integrate both are particularly well-suited to support children's development. Our results indicate that integrated interventions targeting caregiver responsivity and reducing punitive discipline may be particularly effective as both were robustly associated with multiple social-emotional outcomes. Early-life nutritional interventions that include caregiver training may be a cost-effective approach for promoting children's social-emotional competence.

Finally, demographic background characteristics—specifically, child age, child gender, mother's age, maternal depression, and maternal agency—were associated with children's social-emotional outcomes in ways that were highly consistent with prior developmental research. Older children had fewer social-emotional problems across multiple domains while also showing more prosocial behavior, consistent with prior work highlighting age-related increases in emotion regulation and social-emotional competence (73, 97). Boys had more hyperactivity/inattention, but fewer emotional problems and less prosocial behavior compared to girls, consistent with extant literature showing gender differences in social-emotional development for children in LMICs (98). Children with older mothers showed fewer hyperactive/inattentive behaviors and total difficulties, suggesting that greater maternal age may confer some protective benefits.

Notably, mothers with fewer depressive symptoms or who were more self-efficacious had children with fewer social-emotional difficulties across all measured domains and had children who showed greater prosocial behavior. This is consistent with extant research in high-, middle-, and low-income countries demonstrating robust associations between maternal mental health and children's development (99, 100). Considering the intertwined nature of maternal mental health, responsivity, and punitive discipline (99–101), integrated nutritional interventions that also address maternal mental health and caregiving behaviors may optimize both maternal and child social-emotional outcomes. Importantly, meta-analytic evidence suggests that parenting interventions in LMICs aimed at increasing supportive, responsive caregiving did not improve maternal depression, suggesting that parenting behavior and maternal mental health should be treated as distinct targets for interventions in LMICs (95).

### Early-life nutritional supplementation moderates the effects of household physical safety and academic stimulation

Our findings indicate that early-life nutritional supplementation with LNS can play a moderating role in the association between specific home environmental risks and child social-emotional development. In our previous work, we found that children who received LNS were buffered against the adverse effects of overall lower-quality home environments (40), and the present study builds upon this work by identifying which distinct aspects of the home environment quality and which specific social-emotional outcomes are modified by LNS. For children receiving LNS, safer physical environments were associated with fewer difficulties and less hyperactivity/inattention, while household physical safety did not predict outcomes in the control group. Previous studies conducted in North America and Europe have shown that poor housing quality is associated with children's social-emotional difficulties (102, 103), and contributes to psychological distress, mood disorders, and behavioral dysregulation in school-age children from low- and middle-income families (104–106). Our findings suggest that Ghanaian children who received LNS benefitted from physically safer home environments by exhibiting fewer social-emotional problems and were more like their peers in high-income countries. By contrast, children in the control group did not show this same effect, having comparable social-emotional problems regardless of their home's physical safety. This suggests that early-life nutritional supplementation with LNS may enhance the protective effects of a physically safe home.

Interestingly, children who received LNS were buffered against academically under-stimulating households as lower academic stimulation was associated with increased hyperactivity/inattention problems only among children in the control group. As previously discussed, there is robust evidence demonstrating that academic stimulation in the household and children's hyperactivity and attentional problems are negatively associated (82, 84, 88). These findings suggest that nutritional supplementation may differentially protect children against specific environmental risks, particularly physical and academic aspects of their surroundings, potentially reducing the impact of certain adverse conditions on social-emotional outcomes.

### Limitations, strengths, and conclusion

This study was not without limitations. First, the internal consistencies for the SDQ Conduct and Peer Relationship Problems subscales were quite low, which can indicate poor construct reliability (107); however, lower alphas can occur in exploratory research and novel contexts (108, 109). Systematic review of the application and validation of the SDQ in Africa found substantial heterogeneity in alphas between studies, ranging from 0.24 to 0.73 (4), and our alphas fall within that range. Additionally, the mean inter-item correlations for all SDQ subscales exceeded the recommended cut-offs (110). Second, the trial was partially double-blinded as participants were not blinded to whether they received LNS sachets or MMN/IFA tablets during the main trial as the LNS sachets looked different from the MMN/IFA tablets. The MMN/IFA tablets were identical in appearance. The field staff conducting home visits at follow-up and data analysts were blinded. The lack of blinding for mothers may have biased their SDQ responses about their children's behavior as mothers in the LNS group were aware that they were receiving the novel nutritional supplement, which may have led them to have greater expectations for their child's development and may have impacted how they perceived their child's behavior. However, our previous work found no differences between LNS and control groups in parents' perceptions of the positive and negative impacts of the nutritional supplement on children's development at follow-up, suggesting that parents in both the LNS and control groups had equally high expectations (111). Third, two subscales of the newly adapted EC-HOME inventory—physical environment safety and negative behavior acceptance—had only three and four respective test items (see Supplementary material S1, S2), which may lead to low test discrimination and more vulnerability to measurement error.

Despite these limitations, this study has several strengths that enhance its contributions to the literature on child development in LMICs. First, the use of a randomized controlled trial (RCT) design minimizes selection bias and provides robust evidence for causal inferences about the effects of early-life nutritional supplementation. Second, this study also characterized Ghanaian children's home environments by applying factor analysis on test items of a widely used tool, the EC-HOME inventory, in a sample of more than 900 children. The newly adapted EC-HOME inventory showed better internal consistencies of the five new subscales (ranged from 0.43 to 0.79) than those of the original eight subscales (ranged from 0.14 to 0.69). This cultural adaptation of the EC-HOME inventory to the Ghanaian context ensures that the measures used are reliable and reflective of the local household factors influencing child development, enhancing the validity of the findings.

To our knowledge, this is one of the first studies investigating the moderating effect of a nutrition intervention on the association between specific aspects of household quality and both social-emotional strengths and difficulties for children living in an LMIC. By exploring how specific home environment factors interact with early-life nutritional supplementation, this study offers valuable insights into which environmental resources can be incorporated into the design of nutritional interventions, allowing for a more tailored approach to enhancing social-emotional outcomes in LMICs. Future research using a more detailed measure of children's physical environment and a larger sample size of children living in low-quality physical environments may further clarify the interaction of nutrition with the physical environment and several aspects of social-emotional development. A follow-up study of this cohort is needed to investigate whether Ghanaian children's social-emotional difficulties and the relationship between home environment and social-emotional development found in this study persist through school-age and young adulthood. Interventions aiming to reduce punitive discipline, increase caregiver responsiveness, and ensure safe physical environments, alongside nutrition, hold promise for improving social-emotional outcomes and advancing the United Nations' Sustainable Development Goals (UN SDGs) related to health and wellbeing (UN SDG 2, SDG 3). Integrated approaches that align health and psychosocial support can address children's complex developmental needs and may be more effective than isolated interventions alone. Future interventions should consider multipronged approaches that address both nutritional needs and home environment quality to promote early child development and wellbeing while mitigating against environmental risks.

## Data Availability

The raw data supporting the conclusions of this article will be made available by the authors, without undue reservation.
