# Supplementary material for: Early-life nutritional supplementation protects against home environmental risks in Ghanaian children's social-emotional development
Source: Front Nutr. 2025 Aug 20;12:1571677. doi: 10.3389/fnut.2025.1571677 (PMC12404965; doi:10.3389/fnut.2025.1571677)
Supplement: Supplementary file 1 [file Data_Sheet_1.pdf]

S1. Factor Analysis of HOME inventory

The figure S1-1 indicated that the first 15 factors had Eigen values over one. The cumulative variances explained by the first 11 factors are larger than 50%. Each of the 12nd, 13rd, 14th and 15th factors explained below than 2.67% of the variance. Considering the original HOME inventory measure indicated eight factors, we examined solutions for 8, 11 and 15 factors using oblimin rotations and DWLS estimator. The 11 factor solution was preferred because of: (a) more than 50% of variance can be explained; (b) the insufficient number of primary loadings and difficulty of interpreting the 12th factor and the subsequent factors. The table S1-1 showed the factor scores for the HOME inventory items based on the oblimin-rotated 11-factor solution.

Items Removed from the HOME Inventory

A total of six items (items 19, 23, 28, 31, 42, and 43) were eliminated because they did not meet a minimum criteria of having a primary factor loading of 0.3 or above. We also excluded the three factors with only two items in each of the following factors:

1. *Choose Food Factor:* item 18 (Child is permitted choice in breakfast or lunch menu) and item 29 (Parent lets child choose certain favorite food products or brands at the market or store.
2. *Encourage Factor:* item 17 (Parent encourages child to talk and takes time to listen) and item 30 (Parent encourages child to put away toys without help).
3. *No Punishment Factor:* item 24 (Child can express negative feelings without being shouted at) and item 25 (Child can hit parent without being punished or shouted at).

Items Moved to a Different Subscale on the HOME Inventory

Six items had a higher factor loading on a different subscale than the original EC-HOME subscale, therefore we moved these items to the subscale that was appropriate for our context. This impacted the following items and original subscales:

1. *Language Stimulation:* Two items originally in the *Language Stimulation* subscale (10. Child is encouraged to learn the alphabets. 11. Parent teaches child simple verbal manners) were moved to the *Academic Stimulation* subscale. A third item (36. Parent's voice conveys positive feelings about child) was moved to the *Caregiver Responsivity* subscale.
2. *Learning Materials:* One item originally in the *Learning Materials* subscale (16. Child is encouraged to learn shapes (has toys designed for that or caregiver teaches him/her) was moved to the *Academic Stimulation* subscale.
3. *Family Lifestyle Variety:* One item originally in the *Family Lifestyle Variety* subscale (26. Child has real or toy musical instrument) was moved to the *Learning Materials* subscale. A second item (27. Child is taken on outing by a family member at least once every other week) was moved to the *Caregiver Responsivity* subscale

Original Subscales Removed from the Adapted HOME Inventory

After excluding and re-arranging items, no items were left in these three original subscales: *Family Lifestyle Variety*, *Desirable Behavior Modeling*, and *Language Stimulation*. Thus, we removed those original subscales, yielding a final 5-factor structure with the following adapted subscales: *Learning Materials, Physical Environment, Caregiver Responsivity, Academic & Language Stimulation, and Negative Behavior Acceptance.*

A confirmatory factor analysis showed that the adapted 5-factor model fit our data well (χ^2 (496) = 921.98, CFI = 0.97, RMSEA = 0.04). The item description, the original 8-factor structure and the adapted 5-factor structure are shown in Table S1-2.

Figure S1-1.

Scree Plot of the Eigenvalues of Factors for 46 HOME Inventory Items

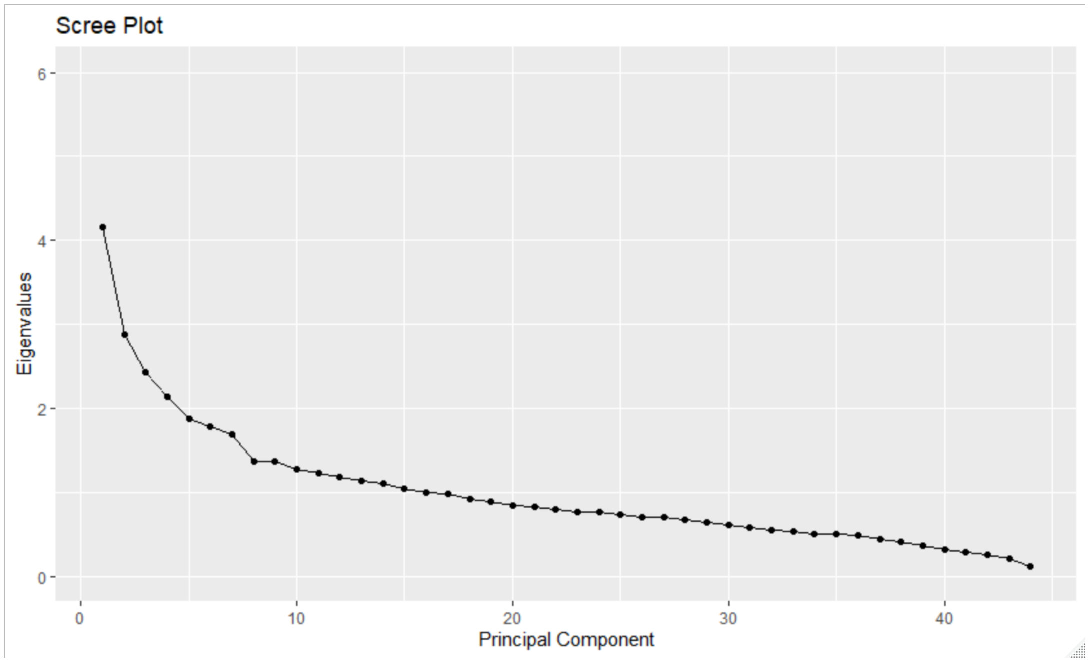

Table S1-1

Factor scores for HOME inventory items based on the oblimin-rotated 11-factor solution

Item	Learning Material	Learning Material 2	Academic + Language	Choose Food	Encourage	No Punishment	Physical environment	Responsivity	Responsivity 2	Acceptance	Acceptance 2
1	**0.72**	0.07	-0.05	0.18	-0.31	0.23	0.22	0.11	0.13	-0.34	0.08
2	**0.59**	0.30	0.04	0.07	-0.28	0.11	-0.10	0.17	0.06	-0.26	-0.06
3	**0.51**	0.14	0.02	-0.41	0.30	-0.12	0.11	-0.14	0.07	-0.22	-0.21
4	**0.54**	0.15	-0.04	0.10	0.02	-0.04	0.09	-0.29	-0.03	0.07	-0.05
5	**0.97**	0.02	0.14	-0.01	0.06	-0.01	-0.13	-0.04	0.01	0.14	0.01
6	**0.98**	-0.04	0.01	-0.02	0.07	0.04	-0.01	0.06	0.05	0.06	-0.01
7	**0.30**	0.24	0.14	-0.09	0.18	-0.05	0.16	-0.10	0.05	-0.30	0.15
8	0.14	**0.70**	0.05	0.08	0.15	-0.07	0.21	-0.11	0.00	-0.05	0.16
9	-0.01	**0.73**	0.18	-0.08	-0.15	-0.01	0.04	0.14	0.06	0.17	-0.02
10	-0.01	0.03	**0.95**	-0.13	0.04	-0.06	-0.01	-0.14	-0.05	-0.11	-0.10
11	0.22	-0.19	**0.47**	-0.38	0.10	-0.18	0.43	0.35	0.00	0.13	0.07
12	0.12	-0.04	**0.81**	0.17	-0.04	0.11	0.16	-0.03	0.03	0.10	0.13
13	0.05	0.13	**0.72**	0.09	-0.12	0.00	0.03	0.18	-0.09	0.28	-0.18
14	-0.02	-0.05	**0.43**	0.01	0.09	-0.05	0.11	0.47	-0.12	-0.05	0.27
15	-0.01	0.18	**0.92**	-0.11	0.08	-0.07	-0.14	0.03	0.00	-0.14	0.15
16	0.20	-0.12	**0.59**	0.18	-0.10	0.18	0.05	0.14	-0.04	0.14	0.10
17	-0.01	-0.01	-0.01	0.02	**0.82**	0.17	0.13	0.07	0.06	-0.03	-0.06
18	-0.02	0.11	0.04	**0.46**	0.35	-0.01	0.05	-0.12	0.04	-0.01	-0.03
19	-0.10	0.08	-0.01	0.01	0.00	-0.08	0.03	-0.06	0.01	0.06	-0.12
22	0.17	0.10	-0.23	0.19	0.31	-0.01	-0.04	**0.34**	0.04	0.30	0.14
23	0.19	0.12	-0.01	0.17	0.28	-0.12	0.29	0.03	-0.08	0.03	0.27
24	0.13	0.00	-0.07	0.00	0.07	**0.83**	-0.01	-0.05	-0.10	0.06	0.10
25	-0.01	-0.04	0.00	0.00	0.05	**0.95**	-0.03	-0.03	-0.10	-0.02	0.04
26	**0.31**	0.14	0.15	0.30	0.06	-0.07	0.09	-0.10	-0.02	0.19	-0.04

27	-0.11	0.22	0.16	-0.08	0.22	0.20	0.13	**0.40**	-0.03	-0.25	-0.33
28	0.17	0.08	-0.07	0.00	-0.02	0.18	0.21	0.09	-0.10	-0.17	-0.25
29	0.17	-0.08	-0.17	**0.47**	0.07	-0.03	0.09	0.15	0.18	-0.16	0.06
30	0.23	-0.18	0.34	0.35	**0.37**	-0.06	-0.09	0.12	0.08	-0.17	-0.01
31	0.26	0.08	-0.12	-0.09	0.21	0.04	0.02	-0.23	-0.21	0.27	-0.34
32	-0.08	-0.03	0.19	-0.07	0.18	0.28	-0.13	-0.08	**0.38**	-0.15	0.24
33	-0.06	-0.02	-0.05	-0.18	0.04	0.04	**0.93**	-0.01	0.06	-0.03	0.14
34	-0.06	0.07	-0.03	-0.05	0.04	0.10	**0.90**	-0.07	0.04	-0.01	-0.09
35	-0.03	0.07	0.01	0.22	0.00	-0.14	**0.96**	0.01	0.00	0.02	-0.12
36	-0.04	-0.01	0.18	-0.02	0.02	0.22	0.21	-0.63	**0.33**	0.15	-0.09
37	0.24	-0.14	-0.07	-0.08	0.10	-0.22	0.14	0.07	**0.73**	0.11	0.04
38	0.17	-0.23	0.03	0.03	-0.12	0.04	0.00	-0.02	**0.84**	0.01	-0.10
39	-0.07	0.18	-0.10	0.05	0.07	-0.12	0.03	-0.09	**1.03**	-0.09	0.06
40	-0.17	0.16	0.24	-0.13	0.00	0.31	0.03	0.24	**0.56**	0.25	-0.21
41	0.07	0.08	-0.17	0.15	0.20	-0.04	-0.04	**0.48**	0.28	0.22	-0.15
42	0.02	0.06	0.18	0.18	-0.02	0.27	-0.02	0.16	0.11	0.16	-0.22
43	0.11	-0.01	-0.03	0.26	0.01	0.03	0.15	0.18	-0.09	0.14	-0.01
44	0.15	0.12	-0.10	-0.04	-0.12	0.26	0.03	-0.09	-0.04	**0.62**	0.45
45	0.01	0.01	-0.03	-0.02	-0.03	0.15	-0.09	0.04	0.05	0.07	**0.94**
46	-0.05	0.04	0.09	0.02	0.01	-0.02	0.03	0.00	-0.04	-0.02	**1.00**

Note. Boldface indicates the highest factor loading of each item and it is larger than 0.3. The two items (items 20 and 21) were not included into the EFA analysis because these two items were used to calculate a variable which indicates whether or not children lived in a crowded environment. The correlation between this crowded item and all other items measuring physical environment is lower than 0.1.

Table S1-2

Items description of the HOME inventory, and the original and the improved factor structure

Item	Item description	Original 8-factor Structure	New 5 factor Structure
1	Child has toys or posters which teach colors, sizes and shapes (FW: Ask which toy it is. Give credit when child has at least two of these toys)	Learning Materials	Learning Materials
2	Child has 1 or more puzzles (Make sure all the pieces are there before you give credit)	Learning Materials	Learning Materials
3	Child has access to or has toys or games in the home permitting free expression (Child must have at least two different types of toys or materials e.g crayon, pencil, paper)	Learning Materials	Learning Materials
4	Child has toys or games requiring refined movements (e.g train set, car, doll with dress that can be removed)	Learning Materials	Learning Materials
5	Child has toys or games or posters which help teach numbers (Having books alone is not enough. Books plus one of the other types of toys should receive credit)	Learning Materials	Learning Materials
6	Child has toys or games or posters which help teach letters/ alphabets (Having books alone is not enough. Books plus one of the other types of toys should receive credit)	Learning Materials	Learning Materials
7	Child has at least 1 children's book at home (must be in readable condition)	Learning Materials	Learning Materials
8	At least 1 book is visible in the home (excluding Bible or Quran)(Observe/Interview)	Learning Materials	Learning Materials

9	Parent or other adults in the home buy or read newspapers or magazines regularly	Learning Materials	Learning Materials
10	Child is encouraged to learn the alphabets	Language Stimulation	Academic & language stimulation
11	Parent teaches child simple verbal manners (please, thank you, I'm sorry)	Language Stimulation	Academic & language stimulation
12	Child is encouraged to learn colors	Academic Stimulation	Academic & language stimulation
13	Child is encouraged to learn patterned speech (e.g nursery rhymes, TV adverts)	Academic Stimulation	Academic & language stimulation
14	Child is encouraged to learn spatial relationships (up/down, left/right)	Academic Stimulation	Academic & language stimulation
15	Child is encouraged to learn numbers	Academic Stimulation	Academic & language stimulation
16	Child is encouraged to learn shapes (has toys designed for that or caregiver teaches him/her)	Learning Materials	Academic & language stimulation
17	Parent encourages child to talk and takes time to listen	Language Stimulation	Excluded
18	Child is permitted choice in breakfast or lunch menu	Language Stimulation	Excluded
19	Child waits until meal times to eat (does not snack anytime he/she is hungry, there is a rule to wait for meal times before eating)	Desirable Behavior Modeling	Excluded
20	How many people are there in this house or what you consider your household who sleep here?	Physical Environment	Excluded
21	How many rooms are there for sleeping in the home (including sitting room or another room)?	Physical Environment	Excluded
22	Parent holds child close 10-15 minutes per day	Caregiver Responsivity	Caregiver Responsivity

23	TV is used judiciously (TV is not left on without control but there are times for watching and certain types of programs that the child is allowed to watch)	Desirable Behavior Modeling	Excluded
24	Child can express negative feelings without being shouted at	Desirable Behavior Modeling	Excluded
25	Child can hit parent without being punished or shouted at	Desirable Behavior Modeling	Excluded
26	Child has real or toy musical instrument	Family Lifestyle Variety	Learning Materials
27	Child is taken on outing by a family member at least once every other week (Anyplace outside of the home e.g friend’s house)	Family Lifestyle Variety	Caregiver Responsivity
28	Child has been on a trip more than 50 miles during the past year (A place that needs transportation to travel to)	Family Lifestyle Variety	Excluded
29	Parent lets child choose certain favorite food products or brands at the market or store	Family Lifestyle Variety	Excluded
30	Parent encourages child to put away toys without help	Family Lifestyle Variety	Excluded
31	Child eats at least one meal on most days with mother and father	Family Lifestyle Variety	Excluded
32	No more than one instance of physical punishment during past week	Acceptance	Acceptance
33	Building appears safe and free of hazards (e.g open gas fires in small rooms, insect or animal traps; frayed electrical cords; sharp kitchen utensils within easy reach of child)	Physical Environment	Physical Environment
34	Outside play environment appears safe	Physical Environment	Physical Environment
35	House is reasonably clean and minimally cluttered	Physical Environment	Physical Environment
36	Parent's voice conveys positive feelings about child	Language Stimulation	Caregiver Responsivity

37	Parent converses with child at least twice during visit	Caregiver Responsivity	Caregiver Responsivity
38	Parent answers child's questions or requests verbally	Caregiver Responsivity	Caregiver Responsivity
39	Parent usually responds verbally to the child's speech	Caregiver Responsivity	Caregiver Responsivity
40	Parent praises child's qualities twice during visit	Caregiver Responsivity	Caregiver Responsivity
41	Parent hugs, kisses, or holds child during visit	Caregiver Responsivity	Caregiver Responsivity
42	Parent helps child demonstrate some achievement during visit	Caregiver Responsivity	Excluded
43	Parent introduces Visitor to child	Desirable Behavior Modeling	Excluded
44	Parent does not scold or yell at or scare or threaten child more than once	Acceptance	Acceptance
45	Parent does not use physical restraint during visit	Acceptance	Acceptance
46	Parent neither slaps nor spanks child during visit	Acceptance	Acceptance

Table S2

Test-retest Reliability and Internal Reliability of original HOME subscales, improved HOME subscales, and SDQ scales

**HOME Inventory**	**Cronbach's Alpha**	**Mean Item-total Correlation**
Original 8 subscales	n = 962	n = 962
Caregiver Responsivity	0.44	0.49
Negative Behavior Acceptance	0.47	0.66
Learning Materials	0.69	0.52
Family Lifestyle Variety	0.23	0.45
Physical Environment	0.58	0.69
Language Stimulation	0.14	0.45
Academic Stimulation	0.54	0.64
Desirable Behavior Modeling	0.27	0.50
Improved 5 subscales		
Caregiver Responsivity	0.43	0.45
Negative Behavior Acceptance	0.47	0.66
Learning Materials	0.69	0.52
Physical Environment	0.79	0.83
Academic & Language Stimulation	0.62	0.55
SDQ		
Emotional Problems	0.73	0.59
Conduct Problems	0.70	0.53
Hyperactive/Inattention	0.72	0.58
Peer Relationship Problems	0.66	0.51
Prosocial Behaviors	0.77	0.68
Total difficulties Score	0.68	0.35

Table S3

Comparison of Participant Characteristics of the Included Children vs. the Excluded Children

Variable	Included Participants<br>Mean ± SD [n] or % [n/total]	Excluded Participants<br>Mean ± SD [n] or % [n/total]	p-value
	n = 962	*n* = 362	
LNS group (%)	35.09% [338/962]	28.73% [104/362]	0.03
Male (%)	47.92% [461/962]	53.10% [154/290]	0.12
Maternal Education (y)	7.61 ± 3.52	7.72 ± 3.95	0.63
Mother Age (y)	26.80 ± 5.42	26.42 ± 5.74	0.26
Maternal Depression	5.74 ± 5.72	6.00 ± 8.72	0.94
Maternal Agency	24.89 ± 4.87	21.60 ± 1.14	0.13
Household asset score(*100) [1]	1.29 ± 95.94	-2.58 ± 110.33	0.62

[1]Proxy indicator for household socioeconomic status constructed for each household based on ownership of a set of assets (radio, television etc.), lighting source, drinking water supply, sanitation facilities, and flooring materials. Household ownership of this set of assets is combined into an index (with a mean of zero and standard deviation of one) using principal components analysis. Higher value represents higher socioeconomic status.

Zero-Order Correlations

We performed zero-order correlations between participants' demographic characteristics, EC-HOME subscale raw scores, and SDQ subscale raw scores for the entire sample (Supplemental Table S4). Nutritional supplement group was negatively correlated with household index asset, suggesting that those who received LNS had fewer household assets. Nutritional supplement group was not significantly correlated with any other background characteristics, SDQ subscale, or EC-HOME score (p's > .05). As expected, all SDQ subscales were significantly correlated with each other (p's < .05). The domains of children's social-emotional problems (i.e., Emotional, Conduct, Hyperactivity/Inattention, Peer Relationship Problems and Total Difficulties) were all positively correlated with each other, suggesting that children scoring higher on one type of social-emotional difficulty were likely to score higher on the others. Conversely, social-emotional difficulties were negatively correlated with Prosocial Behavior, suggesting that children with greater overall social-emotional difficulties exhibited fewer prosocial behaviors.

Also as expected, multiple EC-HOME subscale scores – but not all - were significantly correlated with each other, suggesting that households that were higher quality in one specific aspect of household environment were likely to be higher quality in multiple aspects of household environment. Several significant correlations were observed between social-emotional difficulties and caregiver or household characteristics, conceptually suggesting that children with more social-emotional difficulties reside in lower quality households with comparatively fewer resources (e.g., educational materials), face more punitive discipline, or live in a less physically safe environment. Similarly, Prosocial Behavior positively correlated EC-HOME measures (e.g.,

Caregiver Responsivity, Learning Materials), suggesting that children who were more prosocial may be living in more responsive, higher-quality households.

Finally, there were correlations between background characteristics and key study variables. Maternal Depression positively correlated with children's social-emotional problems, suggesting that maternal and child well-being are associated. Maternal and household advantages (e.g., higher Maternal Education, older mothers, more household assets, higher Maternal Agency) correlated with fewer child difficulties and greater prosocial behaviors. In general, older children showed fewer social-emotional difficulties, more prosocial behavior, had greater access to learning materials, and were more likely to reside in households with lower asset scores and lower maternal education. Girls had more emotional problems and more prosocial behavior.

Table S4.

Zero-order Correlations Between Key Study Variables

Variable	1	2	3	4	5	6	7	8	9	10	11	12	13	14	15	16	17	18	19
1. Nutritional Supplement Group$_A$	-																		
2. Emotional Problems	-.02	-																	
3. Conduct Problems	-.05	.28***	-																
4. Hyperactivity & Inattention	-.01	.10**	.27***	-															
5. Peer Relationship Problems	-.06†	.19***	.21***	.09**	-														
6. Total Difficulties	-.06†	.67***	.69***	.59***	.55***	-													
7. Prosocial Behavior	-.01	-.10**	-.29***	-.20***	-.26***	-.33***	-												
8. Caregiver Responsivity	.01	-.06†	-.15***	-.13***	-.07	-.16***	.18***	-											
9. Negative Behavior Acceptance	.04	-.11**	-.17***	-.15***	-.19***	-.24***	.09**	.04	-										
10. Learning Materials	-.01	-.15***	-.16***	-.15***	-.08*	-.22***	.18***	.19***	.09*	-									
11. Physical Safety	.02	-.16***	-.10**	-.07	-.06	-.16***	.11**	.19***	-.04	.02	-								
12. Academic & Language Stimulation	.01	.06†	-.06†	-.11***	-.07*	-.06†	.07*	.13***	.11**	.01	.02	-							
13. Child Age (y)	.05†	-.19***	-.11**	-.02	-.09**	-.16**	.10***	.05	-.09**	.13***	.29***	.01	-						
14. Child Gender$_B$	.01	-.07*	.03	.06†	-.02	-.01	-.07*	.03	-.05	.03	.03	.01	.05†	-					
15. Mother Age (y)	.01	-.02	-.01	-.13***	-.02	-.07*	.08*	.01	.10**	.05	.03	.01	-.02	.01	-				
16. Maternal Education (y)	.01	-.08**	-.09**	-.08**	-.04	-.12***	.10**	.01	.11**	.08	.36***	.06†	-.08**	.01	.01	-			
17. Maternal Depression	.04	.38***	.21***	.15***	.11**	.35***	-.15***	.04	-.07	.08	-.18***	.04	-.17***	-.02	-.02	-.12***	-		
18. Maternal Agency	.03	-.16***	-.15***	-.16***	-.10**	-.23***	.12***	.03	.05	.13***	.16***	.03	.-02	.01	.01	.06†	-.19***	-	

Variable	1	2	3	4	5	6	7	8	9	10	11	12	13	14	15	16	17	18	19
19. Household Asset Index	-.07*	-.04	-.03	-.09**	-.03	.08*	-.06†	-.07	.11**	.06	.23***	-07†	-.23***	.01	.01	.13***	-.02	-.02	-

Note: N = 962. A. Nutritional Supplement Group was dummy coded (1 = LNS Group, 0 = Control Group). B. Child Gender was dummy-coded (1 = Male, 0 = Female). Child Age, Mother Age, and Maternal Education measured in years. † $p < .10$, * $p < .05$, ** $p < .01$, *** $p < .001$.

Table S5

Unstandardized Covariance Estimates for Early-Life Nutritional Supplementation and Home Environment Factors Predicting to Ghanaian Children's Social-Emotional Strengths & Difficulties

	Model 1: Emotional Problems	Model 2: Conduct Problems	Model 3: Hyperactivity & Inattention	Model 4: Peer Relationship Problems	Model 5: Total Difficulties	Model 6: Prosocial Behavior
Covariances	*b* (SE)	*b* (SE)	*b* (SE)	*b* (SE)	*b* (SE)	*b* (SE)
Nutritional Supplement Group$_A$ ↔						
Child Age	0.02 (0.01)*	0.02 (0.01)*	0.02 (0.01)*	0.02 (0.01)*	0.02 (0.01)*	0.02 (0.01)*
Household Assets Index	-0.04 (0.01)**	-0.04 (0.01)**	-0.04 (0.01)**	-0.04 (0.01)**	-0.04 (0.01)**	-0.04 (0.01)**
Academic & Language Stimulation ↔						
Caregiver Responsivity	0.21 (0.06)***	0.21 (0.06)***	0.21 (0.06)***	0.21 (0.06)***	0.21 (0.06)***	0.21 (0.06)***
Negative Behavior Acceptance	0.12 (0.04)**	0.12 (0.04)**	0.12 (0.04)**	0.12 (0.04)**	0.12 (0.04)**	0.12 (0.04)**
Learning Materials	0.75 (0.09)***	0.75 (0.09)***	0.75 (0.09)***	0.75 (0.09)***	0.75 (0.09)***	0.75 (0.09)***
Physical Safety	0.12 (0.04)**	0.12 (0.04)**	0.12 (0.04)**	0.12 (0.04)**	0.12 (0.04)**	0.12 (0.04)**
Maternal Depression	0.60 (0.22)**	0.58 (0.22)**	0.58 (0.22)**	0.59 (0.22)**	0.58 (0.22)**	0.59 (0.22)**
Maternal Education	0.84 (0.14)***	0.84 (0.14)***	0.84 (0.14)***	0.84 (0.14)***	0.84 (0.14)***	0.84 (0.14)***
Household Assets Index	0.22 (0.04)***	0.22 (0.04)***	0.22 (0.04)***	0.22 (0.04)***	0.22 (0.04)***	0.22 (0.04)***
Field Staff 3	0.05 (0.01)***	0.05 (0.01)***	0.05 (0.01)***	0.05 (0.01)***	0.05 (0.01)***	0.05 (0.01)***
Caregiver Responsivity ↔						
Maternal Depression	-0.52 (0.25)*	-0.51 (0.25)*	-0.53 (0.25)*	-0.53 (0.25)*	-0.55 (0.25)*	-0.52 (0.25)*
Maternal Education	0.52 (0.16)**	0.52 (0.16)**	0.52 (0.16)**	0.52 (0.16)**	0.53 (0.16)**	0.51 (0.16)**
Child Age	-0.07 (0.03)*	-0.07 (0.03)*	-0.07 (0.03)*	-0.07 (0.03)*	-0.07 (0.03)*	-0.07 (0.03)*
Household Assets Index	0.14 (0.04)**	0.14 (0.04)**	0.14 (0.04)**	0.14 (0.04)**	0.14 (0.04)**	0.14 (0.04)**
Learning Materials	0.50 (0.09)***	0.50 (0.09)***	0.50 (0.09)***	0.50 (0.09)***	0.50 (0.09)***	0.50 (0.09)***
Physical Safety	0.21 (0.04)***	0.21 (0.04)***	0.21 (0.04)***	0.21 (0.04)***	0.21 (0.04)***	0.21 (0.04)***
Mother Age	0.62 (0.24)*	0.62 (0.24)*	0.62 (0.24)*	0.62 (0.24)*	0.63 (0.24)*	0.62 (0.24)*
Learning Materials ↔						
Negative Behavior Acceptance	0.09 (0.06)	0.09 (0.06)	0.09 (0.06)	0.09 (0.06)	0.09 (0.06)	0.08 (0.06)
Maternal Depression	-2.08 (0.37)***	-2.07 (0.37)***	-2.07 (0.37)***	-2.07 (0.37)***	-2.08 (0.37)***	-2.08 (0.37)***
Maternal Agency	1.22 (0.31)***	1.21 (0.31)***	1.21 (0.31)***	1.21 (0.31)***	1.21 (0.31)***	1.22 (0.31)***
Maternal Education	2.53 (0.24) ***	2.53 (0.24) ***	2.53 (0.24) ***	2.53 (0.24) ***	2.53 (0.24) ***	2.53 (0.24) ***
Child Age	0.14 (0.03) ***	0.14 (0.03) ***	0.14 (0.03) ***	0.14 (0.03) ***	0.14 (0.03) ***	0.14 (0.03) ***
Household Assets Index	0.42 (0.06)***	0.42 (0.06)***	0.42 (0.06)***	0.42 (0.06)***	0.42 (0.06)***	0.42 (0.06)***
Physical Safety	0.36 (0.06)***	0.36 (0.06)***	0.36 (0.06)***	0.36 (0.06)***	0.36 (0.06)***	0.36 (0.06)***

Field Staff 1	-0.12 (0.02)***	-0.12 (0.02)***	-0.12 (0.02)***	-0.12 (0.02)***	-0.12 (0.02)***	-0.12 (0.02)***
Field Staff 3	0.08 (0.02)***	0.08 (0.02)***	0.08 (0.02)***	0.08 (0.02)***	0.08 (0.02)***	0.08 (0.02)***
Negative Behavior Acceptance ↔						
Maternal Depression	0.42 (0.17)**	0.45 (0.17)**	0.44 (0.17)**	0.44 (0.17)**	0.44 (0.17)**	0.44 (0.17)**
Maternal Agency	0.53 (0.15)**	0.52 (0.15)***	0.54 (0.15)***	0.54 (0.15)***	0.54 (0.15)***	0.53 (0.15)***
Child Gender$_B$	-0.04 (0.02)**	-0.04 (0.02)**	-0.04 (0.02)**	-0.04 (0.02)**	-0.04 (0.02)**	-0.04 (0.02)**
Field Staff 2	0.03 (0.01)*	0.03 (0.01)*	0.03 (0.01)*	0.03 (0.01)*	0.03 (0.01)*	0.03 (0.01)*
Field Staff 4	-0.07 (0.01)***	-0.07 (0.01)***	-0.07 (0.01)***	-0.07 (0.01)***	-0.08 (0.01)***	-0.07 (0.01)***
Physical Safety ↔						
Maternal Depression	-1.09 (0.17)***	-1.09 (0.17)***	-1.09 (0.17)***	-1.09 (0.17)***	-1.10 (0.17)***	-1.09 (0.17)***
Maternal Education	0.24 (0.10)*	0.24 (0.10)*	0.24 (0.10)*	0.24 (0.10)*	0.24 (0.10)*	0.24 (0.10)*
Child Age	0.04 (0.02)*	0.04 (0.02)*	0.04 (0.02)*	0.04 (0.02)*	0.04 (0.02)*	0.04 (0.02)*
Household Assets Index	0.13 (0.03)***	0.13 (0.03)***	0.13 (0.03)***	0.13 (0.03)***	0.13 (0.03)***	0.13 (0.03)***
Field Staff 1	-0.02 (0.01)*	-0.02 (0.01)*	-0.02 (0.01)*	-0.02 (0.01)*	-0.02 (0.01)*	-0.02 (0.01)*
Field Staff 3	0.03 (0.01)***	0.03 (0.01)***	0.03 (0.01)***	0.03 (0.01)***	0.03 (0.01)***	0.03 (0.01)***
Maternal Depression ↔						
Maternal Agency	-5.15 (0.89)***	-5.09 (0.89)***	-5.09 (0.89)***	-5.11 (0.89)***	-5.09 (0.89)***	-5.13 (0.89)***
Mother Age	2.94 (0.93)**	2.91 (0.93)**	2.90 (0.93)**	2.92 (0.93)**	2.91 (0.93)**	2.92 (0.93)**
Field Staff 1	0.49 (0.07)***	0.49 (0.07)***	0.49 (0.07)***	0.49 (0.07)***	0.49 (0.07)***	0.49 (0.07)***
Field Staff 4	-0.29 (0.07)***	-0.29 (0.07)***	-0.29 (0.07)***	-0.29 (0.07)***	-0.29 (0.07)***	-0.29 (0.07)***
Field Staff 1 ↔						
Maternal Agency	-0.14 (0.05)**	-0.14 (0.05)**	-0.14 (0.05)**	-0.14 (0.05)**	-0.14 (0.05)**	-0.14 (0.05)**
Household Assets Index	-0.02 (0.01)†	-0.02 (0.01)†	-0.02 (0.01)†	-0.02 (0.01)†	-0.02 (0.01)†	-0.02 (0.01)†
Field Staff 2	-0.05 (0.01)***	-0.05 (0.01)***	-0.05 (0.01)***	-0.05 (0.01)***	-0.05 (0.01)***	-0.05 (0.01)***
Field Staff 3	-0.03 (0.01)***	-0.03 (0.01)***	-0.03 (0.01)***	-0.03 (0.01)***	-0.03 (0.01)***	-0.03 (0.01)***
Field Staff 4	-0.05 (0.01)***	-0.05 (0.01)***	-0.05 (0.01)***	-0.05 (0.01)***	-0.05 (0.01)***	-0.05 (0.01)***
Field Staff 2 ↔						
Field Staff 3	-0.03 (0.01)***	-0.03 (0.01)***	-0.03 (0.01)***	-0.03 (0.01)***	-0.03 (0.01)***	-0.03 (0.01)***
Field Staff 4	-0.05 (0.01)***	-0.05 (0.01)***	-0.05 (0.01)***	-0.05 (0.01)***	-0.05 (0.01)***	-0.05 (0.01)***
Field Staff 3 ↔						
Field Staff 4	-0.03 (0.01)***	-0.03 (0.01)***	-0.03 (0.01)***	-0.03 (0.01)***	-0.03 (0.01)***	-0.03 (0.01)***

Note. N=962. A. Nutritional Supplement Group was dummy coded (1 = LNS Group, 0 = Control Group). B. Child Gender was dummy-coded (1 = Male, 0 = Female). Field Staff distinguishes data collectors. † $p < .10$, * $p < .05$, ** $p < .01$, *** $p < .001$

Figure S2

Covariances Between Nutrition Group, Home Quality, and Covariate Variables for all Path Models

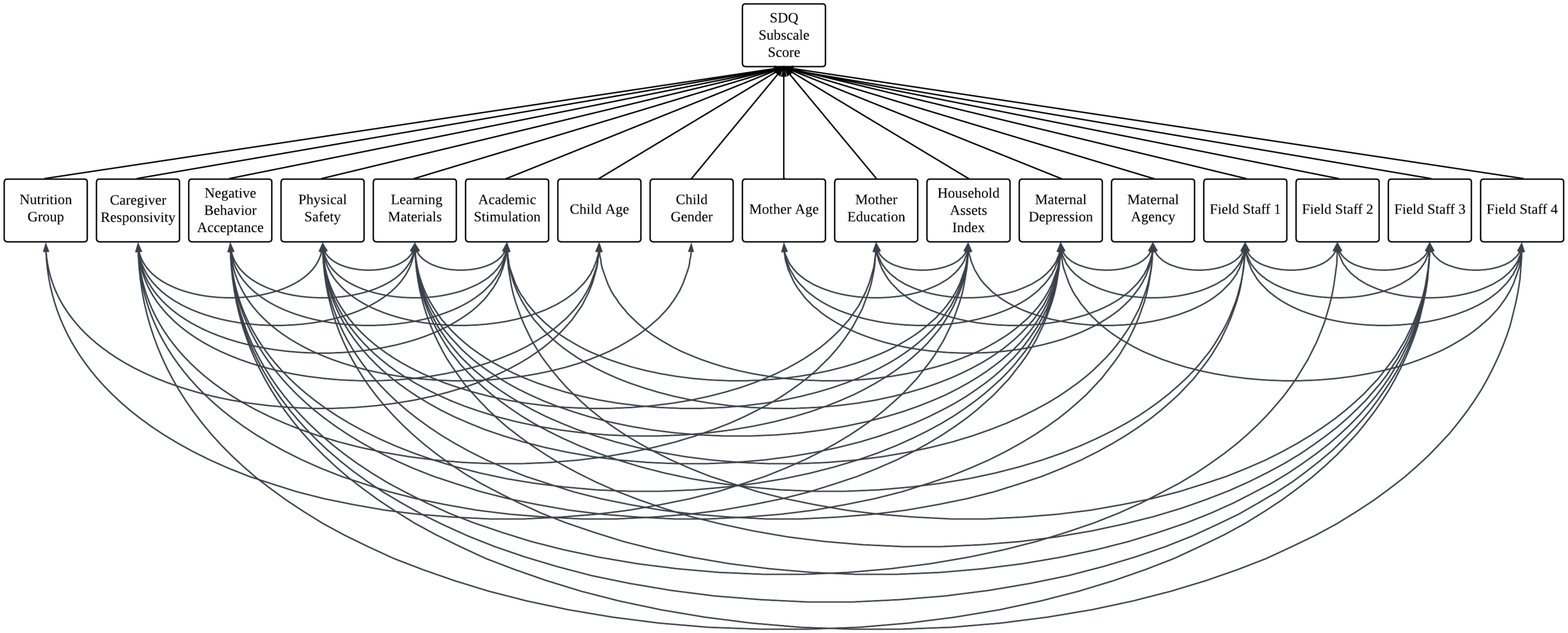

Six path models fit for each of the six SDQ subscale scores. Nutrition Group was dummy-coded (1 = LNS, 0 = Control). Child Gender was dummy-coded (Male = 1, Female = 0). For multi-group comparison analyses (Aim 3), the nutrition group regression path and covariances were removed for all six models.

Post Hoc Power Analyses

Because path models in an SEM framework require specifying a large and complex covariance structure (e.g., regression estimates, variances, covariances, residuals, etc.), conducting a true a priori power calculation for each individual path is not straightforward (1,2). Additionally, standard SEM power calculators often rely on overall fit indices (e.g., testing whether RMSEA ≤ .05 versus > .05), which does not directly translate to detecting each predictor's effect on an outcome (1). Thus, our analytic decisions were guided by widely used guidelines for sample adequacy in structural equation modeling. Specifically, we aimed to meet or exceed the recommended minimum cases-to-parameters ratio (k:1) of 10:1 (3). For our most complex path model, which included 63 freely estimated parameters, our final analytic sample size of 962 yielded a k:1 ratio of approximately 15:1, which supports sufficient power, stable parameter estimates, and model convergence (2).

As a robustness check, we followed the recommendations of Wang and Rhemtulla (2021) and conducted post hoc Monte Carlo simulations for our six key predictors (LNS, Caregiver Responsivity, Negative Behavior Acceptance, Access to Learning Materials, Physical Environment, Academic & Language Stimulation) across each of the six SDQ outcomes (Prosociality, Total Difficulties, Hyperactivity/Inattention, Peer Relationship Problems, Conduct Problems, and Emotional Problems). Using the observed regression coefficients from our sample ($N = 962$) as "population" values, we simulated 1,000 replications of data at $N = 962$, re-fit each model, and recorded the proportion of times each path was statistically significant ($p < .05$). Results are presented in Table S6.

Overall, these simulations further support that our sample of 962 provided adequate power to detect all our primary, theoretically driven paths. Our large sample ($N = 962$) ensures that all medium-to-large effects in the SEM path analyses are well powered. Key predictors (e.g.,

Caregiver Responsivity, Negative Behavior Acceptance) that showed significant associations had ≥ .80 simulated power; nonsignificant associations among those same key predictors (e.g., Responsivity → Emotional Problems; LNS → prosociality) likely reflect genuinely null associations.

Table S6

Post Hoc Power Estimates from Monte Carlo Simulations for Hypothesis Testing Models

	Prosociality			Total Difficulties			Hyperactivity & Inattention			Peer Relationship Problems			Conduct Problems			Emotional Problems		
**Predictor**	***b***	***p*-value**	**Power**	***b***	***p*-value**	**Power**	***b***	***p*-value**	**Power**	***b***	***p*-value**	**Power**	***b***	***p*-value**	**Power**	***b***	***p*-value**	**Power**
LNS	.01	.94	.07	-.59	.02	.61	-.06	.63	.09	-.22	.03	.88	-.20	.07	.44	-.12	.33	.16
Caregiver Responsivity	.19	.00	.99	-.29	.01	.85	-.10	.04	.58	-.03	.40	.27	-.12	.01	.90	-.03	.53	.13
Negative Behavior Acceptance	.07	.33	.18	-.99	<.001	1.00	-.22	.00	.94	-.27	<.001	1.00	-.23	<.001	.98	-.26	.00	.98
Access to Learning Materials	.07	.03	.71	-.15	.04	.55	-.04	.19	.27	.01	.82	.07	-.06	.05	.51	-.05	.15	.33
Physical Environment	.04	.60	.10	-.12	.45	.12	.01	.91	.04	.00	.94	.06	-.02	.79	.06	-.12	.13	.38
Academic & Language Stimulation	.02	.65	.09	-.01	.93	.06	.11	.02	.65	-.02	.61	.13	-.02	.64	.09	.14	.01	.80
